# Tailoring the Nucleation and Crystallization Rate of Polyhydroxybutyrate by Copolymerization

**DOI:** 10.1021/acs.biomac.3c00808

**Published:** 2023-10-02

**Authors:** Maria Rosaria Caputo, Changxia Shi, Xiaoyan Tang, Haritz Sardon, Eugene Y.-X. Chen, Alejandro J. Müller

**Affiliations:** † POLYMAT and Department of Polymers and Advanced Materials: Physics, Chemistry and Technology, Faculty of Chemistry, University of the Basque Country UPV/EHU, Paseo Manuel de Lardizabal 3, 20018 Donostia-San Sebastián, Spain; ‡ Department of Chemistry, Colorado State University, Fort Collins, Colorado 80523-1872, United States; § IKERBASQUE, Basque Foundation for Science, Plaza Euskadi 5, 48009 Bilbao, Spain

## Abstract

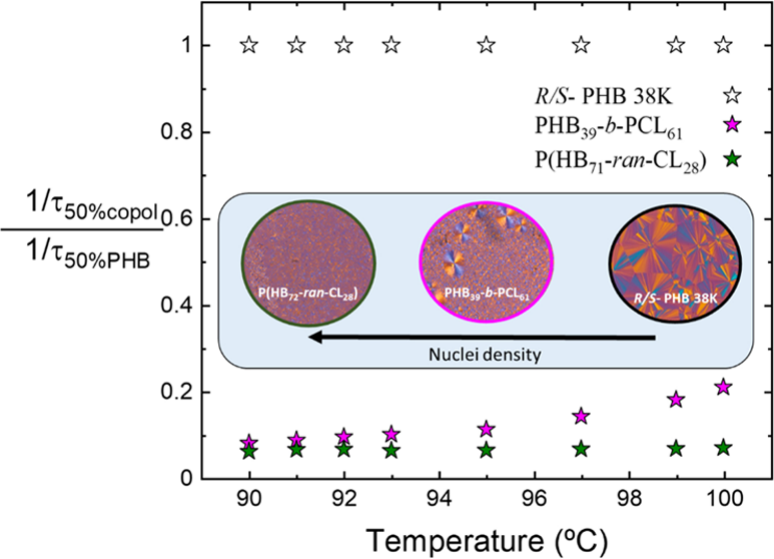

In the polyester family, the biopolymer with the greatest industrial potential could be poly(3-hydroxybutyrate) (PHB), which can be produced nowadays biologically or chemically. The scarce commercial use of PHB derives from its poor mechanical properties, which can be improved by incorporating a flexible aliphatic polyester with good mechanical performance, such as poly(ε-caprolactone) (PCL), while retaining its biodegradability. This work studies the structural, thermal, and morphological properties of block and random copolymers of PHB and PCL. The presence of a comonomer influences the thermal parameters following nonisothermal crystallization and the kinetics of isothermal crystallization. Specifically, the copolymers exhibit lower melting and crystallization temperatures and present lower overall crystallization kinetics than neat homopolymers. The nucleation rates of the PHB components are greatly enhanced in the copolymers, reducing spherulitic sizes and promoting transparency with respect to neat PHB. However, their spherulitic growth rates are depressed so much that superstructural growth becomes the dominating factor that reduces the overall crystallization kinetics of the PHB component in the copolymers. The block and random copolymers analyzed here also display important differences in the structure, morphology, and crystallization that were examined in detail. Our results show that copolymerization can tailor the thermal properties, morphology (spherulitic size), and crystallization kinetics of PHB, potentially improving the processing, optical, and mechanical properties of PHB.

## 1 Introduction

One of the most critical challenges for contemporary society is the need to decrease the use of plastics derived from petroleum sources and promote the production and use of biobased materials. In this context, packaging materials defined as “sustainable” have been identified as priorities by manufacturing industries and consumers.^1,2^ Aliphatic polyesters are a priority, given their biodegradability and biocompatibility.^3−6^ A class of polyesters much studied in the past decade is that of polyhydroxyalkanoates, PHAs,^7−9^ of bacterial origin^10^ and produced in bacterial cytoplasm as a source of carbon and energy storage.^11,12^ Research has demonstrated that PHAs undergo complete degradation in a time span ranging from 6 months to 2 years.^13^ On the other hand, the PHB biodegradation process does not foresee the formation of toxic products and, specifically, it has the capability to occur in both aerobic and anaerobic environments: the products of the aerobic process are carbon dioxide and water, while the products of the anaerobic process are carbon dioxide and methane.^14,15^ The biodegradation of PHB and its copolymers can occur by bacteria and fungi (microorganisms) found in soil or industrial waste. Microorganisms are able to release enzymes (i.e., PHB depolymerase^16^), which are used to degrade polymers up to hydroxy acids, constituent elements of polyhydroxyalkanoates.

Given its thermal properties resembling those of isotactic polypropylene,^17−19^ among the PHA family, PHB is the most extensively researched polymer. PHB has many advantages: it is resistant to humidity and ultraviolet rays, has excellent barrier properties, and is water-insoluble.^20,21^ However, it also has some disadvantages as well: it is highly brittle^22−24^ and thermally decomposes immediately after melting,^25,26^ thus severely limiting its industrial use. One way to spread the use of PHB-based materials is a chemical modification or copolymer formation to improve its hydrophilic character and use it in the biomedical field. In fact, copolymerization with vinyl terminal groups^27^ and with poly(ethylene oxide) (PEO)^28^ is common practice. Another disadvantage of PHB is that its bacterial synthesis is slow and with little control over the molecular weights and, therefore, a synthetic route has recently been developed to produce PHB chemically.^29,30^

Purely isotactic PHB produced from chemical synthesis is not enantiomerically pure *R* as the bacterial one, but it is a racemic mixture (*R*/*S*); its structural and thermal properties have been studied and found to be very similar to those of bacterial PHB.^31^ The isotactic PHB from chemical synthesis also has similarly poor mechanical properties as the PHB of bacterial origin, and therefore, investigations for their improvement have been conducted. Recently, it has been made possible to obtain an interesting and important result: the controlled introduction of stereodefects in semicrystalline PHB chains led to a PHB material with optical and mechanical capabilities comparable to isotactic polypropylene.^32^

Furthermore, a standard approach to enhance the mechanical properties of PHB is through copolymerization with monomers of other PHAs to obtain copolymers, such as poly(3-hydroxybutyrate-*co*-3-hydroxyhexanoate) (PHBH) and poly(3-hydroxybutyrate-*co*-3-hydroxyvalerate) (PHBV). These copolymers are softer and more flexible, and they melt at lower temperatures than neat PHB and have higher impact resistance. The advantage of such materials is that their properties can be tuned according to their composition; however, their disadvantage is that, up to now, their bacterial synthesis does not allow complete control of their composition and steroregularity.^11,33^

Furthermore, one way to improve the fragility of PHB is copolymerization with polyhydroxyoctanoate (PHO), which is an elastic material, and this leads to the formation of flexible packaging materials.^34^

Thus, further steps have been taken to make the production and use of PHB-based materials easier: the path taken in recent years has been to produce blends with PLA^22,35,36^ and PCL,^37−39^ for example. However, in this case, problems due to degradation or the uncontrolled nature of biologically produced PHB remained.

A synthetic route has recently been reported to produce copolymers based on PHB and poly(ε-caprolactone) (PCL).^40^ PCL is a semicrystalline polyester with low glass transition (*T*_g_ = −60 °C) and melting (*T*_m_ = 50–70 °C) temperatures and excellent mechanical properties, as it is ductile even with a high degree of crystallinity.^41^ It is one of the most used polyesters in biomedical and packaging applications, given its biocompatibility and biodegradability.^42−44^ Its biocompatibility is due to the fact that PCL, under physiological conditions, degrades by hydrolysis of its ester bonds.^45,46^ But it can also be biodegraded by microorganisms present in the soil and by fungi.^47,48^ The intuition in choosing PCL, due to its excellent mechanical properties, was successful as the resulting materials were ductile and tough, as they synergistically combine the best properties of the starting materials: the high Young’s modulus of PHB^49^ and the ductility of PCL.^50^

The result of the work carried out by Tang et al.^40^ is two types of new copolymers: a PHB-*b*-PCL block copolymer and a P(HB-*ran*-CL) random copolymer. As both PHB and PCL are semicrystalline materials, it is of utmost importance to study how their structure, nucleation, and crystallization are affected by the incorporation of a second crystallizable comonomer. Regulating the crystallization rate and degree of crystallinity is a determining factor for applications, as the biodegradation rate, permeation, and mechanical properties critically depend on the crystallinity degree and morphology (spherulitic size). Therefore, this work aims to study the structure, morphology, nucleation, and overall crystallization rate of two representative PHB/PCL random and block copolymers compared to their homopolymers.

## 2 Experimental Section

### 2.1 Materials

#### 2.1.1 Standard Copolymerization Methodology

Polymerizations to produce the two copolymer samples were performed in our previous work^40^ and in 100 mL glass reactors inside an inert glovebox at room temperature (∼23 °C). The reactor was filled with a predetermined quantity of monomers (mixture of racemic eight-membered dimethyl diolide, *rac*-8DL^Me^, with ε-caprolactone, ε-CL) and dichloromethane (DCM) in a glovebox, and the mixture of catalyst and initiator in DCM was stirred at room temperature for 10 min in another 5.5 mL reactor. The polymerization was initiated by rapidly adding the catalyst solution to the monomer solution. Once the desired duration elapsed, the polymerization process was promptly quenched by introducing 5 mL of benzoic acid/chloroform (10 mg/mL). Subsequently, 0.02 mL of sample was extracted from the reaction mixture and processed by ^1^H NMR analysis to determine the percentage of monomer conversion. After quenching, the mixture was poured into 300 mL of cold methanol under constant stirring. The precipitate was then filtered, washed with cold methanol to eliminate any remaining unreacted monomers, and finally, it was dried at room temperature in a vacuum oven until a constant weight was achieved. More details and the scheme of reactions are given in the SI, as well as the ^1^H NMR spectra.

#### 2.1.2 Materials for Comparison Purposes

For comparison purposes, homopolymer PCL and PHB samples with molecular weights similar to those of the prepared copolymers were used. The PCL sample was synthesized according to the procedure reported by Fernández-Tena et al.,^51^ and PHB was obtained according to Tang et al.^30,52^ Data for these two comparative samples were obtained by Fernández-Tena et al.^51^ for the PCL and by Caputo et al.^31^ for the PHB. Table 1 reports the molecular weight and dispersity of the materials employed.

**Table 1:** Molecular Weight and Dispersity Values of the Reference Homopolymers and the Two Copolymers Studied in This Work

sample	*M*_n_ (g/mol)^a^	*Đ*
*R*/S PHB-38K	38,000^a^	1.07
PCL-22K	22,100^b^	1.60
PHB_39_-*b*-PCL_61_	36,000^a^	1.01
P(HB_72_-*ran*-CL_28_)	75,000^a^	1.05

a Measured by size-exclusion chromatography (SEC), as described by Tang et al.^52^ in chloroform.

b Measured by SEC, as described by Fernández-Tena et al.^51^

### 2.2 Characterization Methods

To remove any catalyst residue coming from the synthesis, the two copolymer samples involved in this study were purified by dissolving in hot chloroform and then precipitated into methanol and dried under a vacuum at 60 °C for 24 h.

#### 2.2.1 NMR Spectroscopy

^1^H (NMR) spectra were recorded in a Bruker Avance DPX 300 at 300.16 MHz resonance frequency. Samples were dissolved in deuterated chloroform (CDCl_3_) and kept for a few minutes at 60 °C to dissolve. The experimental conditions were as follows: 3 s acquisition time, 1 s delay time, 8.5 μs pulse, spectral width 5000 Hz, and 32 scans. Chemical shifts for all spectra were referenced to internal solvent resonances and were reported as parts per million relative to SiMe_4_.

#### 2.2.2 Thermogravimetric Analysis (TGA)

A PerkinElmer TGA was used to determine the degradation temperature of the copolymers studied in this work. This analysis was also performed to compare them to neat homopolymers.^31,51^ To carry out this experiment, approximately 7 mg of the sample was placed in a platinum crucible and heated to 600 °C at 20 °C/min.

#### 2.2.3 Small Angle X-ray Scattering (SAXS)

SAXS experiments were performed during the crystallization and melting of the samples. These experiments were performed at the BL11-NCD beamline in the ALBA Synchrotron in Barcelona (Cerdanyola del Vallés, Spain).

To carry out the heat treatment, the samples were placed in aluminum pans (the same ones used in the DSC), and a Linkam THMS600 hot-stage was used for controlled crystallization and melting of the materials.

The energy of the X-ray source is 12.4 keV, corresponding to a wavelength of 1A, and the exposure time is 2 s. For the acquisition of the SAXS spectrum, the sample–detector distance was 6640 mm with a tilt angle of 0°, and a Pilatus 1 M was employed as a detector, supplied by Dectris with an active area of 981 × 1043 pixels and a pixel size of 172 μm^2^. Silver behenate was used for the calibration. The SAXS profiles are plotted as a function of the scattering vector *q* (=4π sin(θ)λ^–1^, where λ is the X-ray wavelength and 2θ is the scattering vector).

#### 2.2.4 Differential Scanning Calorimetric Analysis (DSC)

The thermal properties of these copolymers were determined using a PerkinElmer 8000 DSC instrument with an Intracooler 2P, calibrated with tin and indium as standards. To conduct the analysis, 5 mg of the sample was placed in sealed aluminum pans, and isothermal and nonisothermal experiments were performed.

To determine the melting and crystallization temperatures of the materials, nonisothermal experiments were conducted, during which the samples were heated up to 195 °C and left at this temperature for 3 min to erase the thermal history. Subsequently, the samples were cooled at 20 °C/min down to −20 °C and left at this temperature for 1 min. Then, a heating scan was performed up to 195 °C at a rate of 20 °C/min.

Furthermore, isothermal crystallization experiments were conducted to study the overall crystallization kinetics.

In the case of the block copolymer, since both blocks are crystallizable, an isothermal study of the crystallization of both blocks was conducted separately, as shown below. The first step was the determination of the *T*_c,min_, according to the method proposed by Lorenzo et al.,^53^ which is a trial and error method in which the samples are cooled from the melt at 60 °C/min until they reach different *T*_c_ values, then they are heated at 20 °C/min up to 30 °C above the melting temperature of the block under examination (195 °C for the PHB block and 90 °C for the PCL block). If, during the heating step, the material does not show any melting, this indicates that the polymer was not able to crystallize during the cooling to *T*_c_, and thus, this specific *T*_c_ value is suitable for performing isothermal experiments. Then, another *T*_c_ value is chosen, and the procedure is repeated at progressively lower *T*_c_ values until the sample starts to crystallize during cooling at 60 °C/min. Only *T*_c_ values at which the sample does not crystallize during cooling at 60 °C/min can be used for isothermal experiments (see more details in refs (53,54))

The procedure proposed by Müller et al. for the isothermal crystallization experiments was followed.^53,54^ However, since in PHB_39_-*b*-PCL_61_, both blocks are crystallizable, a separate study of the kinetics of each block was performed. At first, the overall crystallization kinetics of the PHB block was investigated (keeping the PCL block molten) and, subsequently, that of the PCL block.

**Figure 1:**
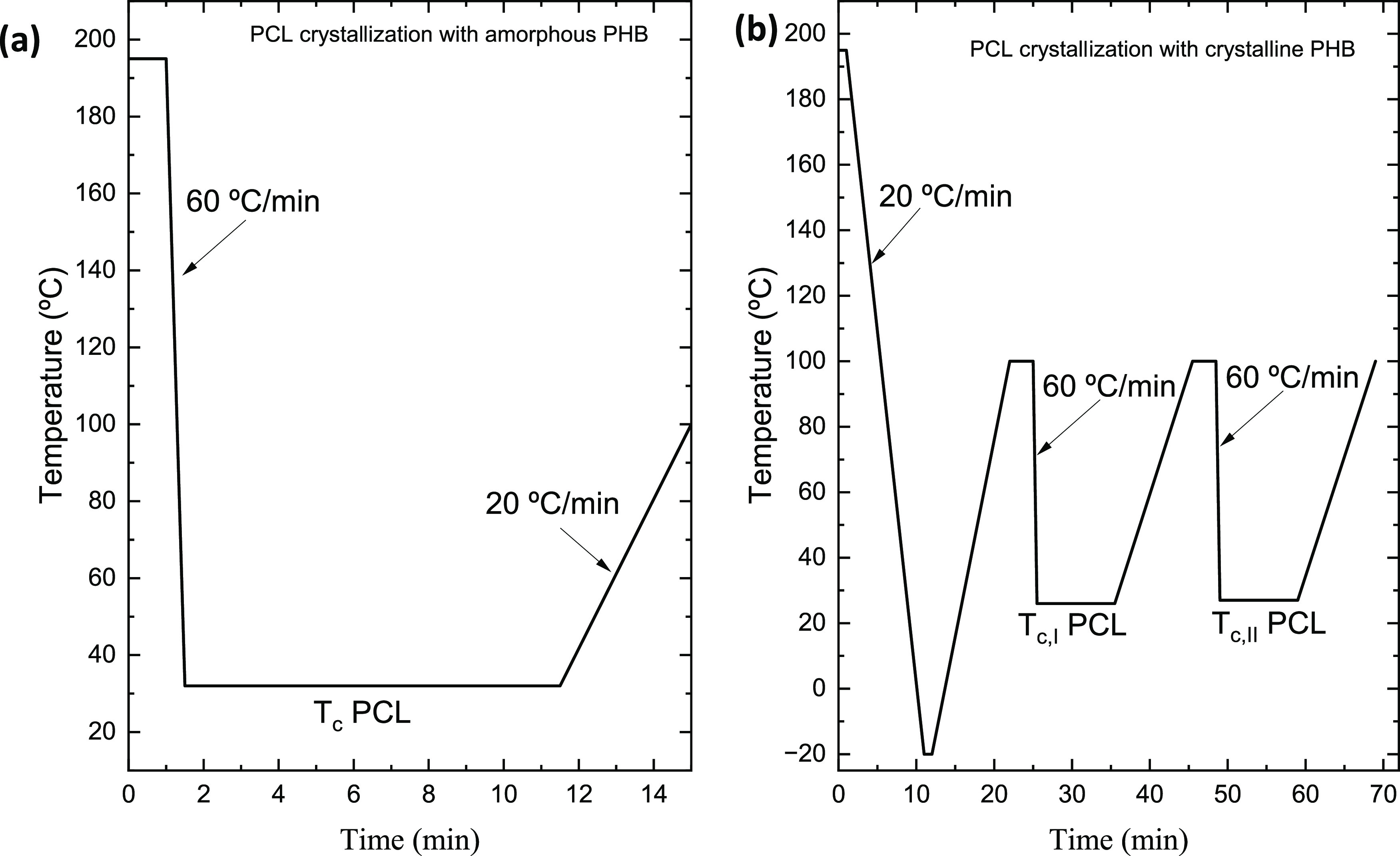
Representative scheme of the procedure for studying the isothermal crystallization kinetics of PCL with amorphous (a) and semicrystalline PHB (b).

The overall crystallization kinetics of the PCL block was studied for two cases: keeping the PHB block amorphous or semicrystalline. In the first case, see Figure 1a, rapid cooling from the melt was performed (at 60 °C/min) to the *T*_c_ chosen for PCL, a condition under which the PHB block could not crystallize.

In the second case, the PHB block was allowed to cold crystallize; see Figure 1b. The sample is first cooled from the melt at 20 °C/min down to −20 °C, then it is heated to 100 °C. During this heating step, the PCL block crystals melt, and soon after, the PBB block undergoes cold crystallization (see also Figure 3 below). At 100 °C, the PHB block has completed its cold-crystallization process. Then, cooling from 100 °C to *T*_c_ is performed at 60 °C/min. In this way, the PHB block crystals remained unmolten, while the PCL isothermal crystallization was determined, as shown in Figure 1b.

In the case of the P(HB_72_-*ran*-CL_28_) sample, since it exhibits only one melting and crystallization, this separate study was not conducted. The procedure for studying isothermal crystallization kinetics involves several steps:^53^ the first, in which the sample is heated from room temperature up to *T*_m_ + 30 °C; and the second, where the sample is left at this temperature for 3 min to erase the thermal history. In the third step, the sample is rapidly cooled (quenched at 60 °C/min) to a chosen crystallization temperature, and in the fourth step, the sample is kept at *T*_c_ for the time needed for crystallization to be saturated. The last step involves heating the crystallized sample from *T*_c_ to its full melting. In this last step, it is normally possible to obtain the experimental melting point of the isothermally crystallized sample, which can be used for the Hoffman–Weeks extrapolation to calculate the equilibrium melting temperature.

The degree of crystallinity was calculated from the DSC second heating scan and during the isothermal step as follows
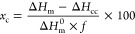
where Δ*H*_m_ is the melting enthalpy, Δ*H*_cc_ is the cold-crystallization enthalpy, Δ*H*_m_^0^ is the enthalpy of fusion at equilibrium (146 J/g for PHB^55^ and 139 J/g for PCL^51^), and *f* is the percentage of copolymer present in each sample.

In the case of calculating the degree of crystallinity as a function of the crystallization temperature for the isothermal crystallization experiments, the isothermal crystallization enthalpy was used.

#### 2.2.5 Polarized Light Optical Microscope Analysis (PLOM)

To study the morphology of the crystallized samples from the melt, an optical microscope with polarized light was used, the Olympus BX51 (Olympus, Tokyo, Japan), using an Olympus SC50 digital camera and a Linkam-15 TP-91 hot stage (Linkam, Tadworth, U.K.) equipped with a liquid nitrogen cooling system. Film samples were prepared in the form of films with a thickness of 50 μm by melting between two glass slides. The samples were first heated up to 190 °C and held for 1 min at this temperature to erase the thermal history, and then they were cooled to room temperature at 20 °C/min.

Experiments with isothermal crystallization were performed to measure the growth rate of spherulites in the PHB-*b*-PCL sample. The sample was molten between two glass slides at 185 °C and left at this temperature for 1 min to erase the thermal history. After that, it was rapidly cooled (50 °C/min) to the crystallization temperatures to allow the appearance of the spherulites, and their isothermal growth was followed as a function of time by taking micrographs. The isothermal crystallization experiments were conducted at various temperatures, and, at each temperature, the radius of the spherulites was measured and recorded over time to determine their growth rate. The Lauritzen–Hoffman equation was used to fit the experimental values.

## 3 Results and Discussion

### 3.1 Melt-Segregation by In Situ SAXS Real-Time Synchrotron

Diblock copolymers can undergo phase separation, and this can be anticipated by evaluating the segregation strength, denoted by the product χ*N*, where χ is the Flory–Huggins interaction parameter, and *N* is the degree of polymerization.

The equation^56^ below can be used to calculate an approximate value of the Flory–Huggins interaction parameter (χ)
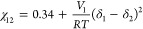
where *V*_1_ corresponds to the molar volume of component 1, *T* (K) represents the temperature at which both polymers are in the molten state (468 K or 195 °C), *R* is the gas constant with the value of 1.987 cal/K, and δ_1_ and δ_2_ are the solubility parameters of each block expressed in (cal/cm^3^)^1/2^.

In this case, to calculate the interaction parameter χ, a reference molar volume of 100 cm^3^/mol was employed, and the solubility parameters for each block were obtained from existing literature sources: [δ_PHB_ = 9.14 (cal/cm^3^)^1/2^; δ_PCL_ = 9.39 (cal/cm^3^)^1/2^].^56,57^ Subsequently, the value of product χ*N* was calculated and turned out to be approximately 33. For block copolymers, there are different degrees of miscibility in the melt based on the value that the product χ*N* assumes: when χ*N* is ≤10, the blocks in the copolymer are miscible in the melt, when χ*N* is between 10 and 30, the blocks are weakly segregated, when χ*N* is between 30 and 50 the blocks are intermediately segregated, and, finally, when χ*N* is >50 the two blocks are strongly segregated in the melt. Consequently, in the case of the system under examination, the separation that occurs is intermediate, as demonstrated also by the presence of the spherulites analyzed below (a fact that indicates that the phase segregation was overcome by the crystallization that was able to break out of the constraints of the phase-segregated domains).

To better understand this phase separation in the melt, SAXS experiments were performed in the block copolymer, the spectrum of which was compared with that of the random copolymer.

**Figure 2:**
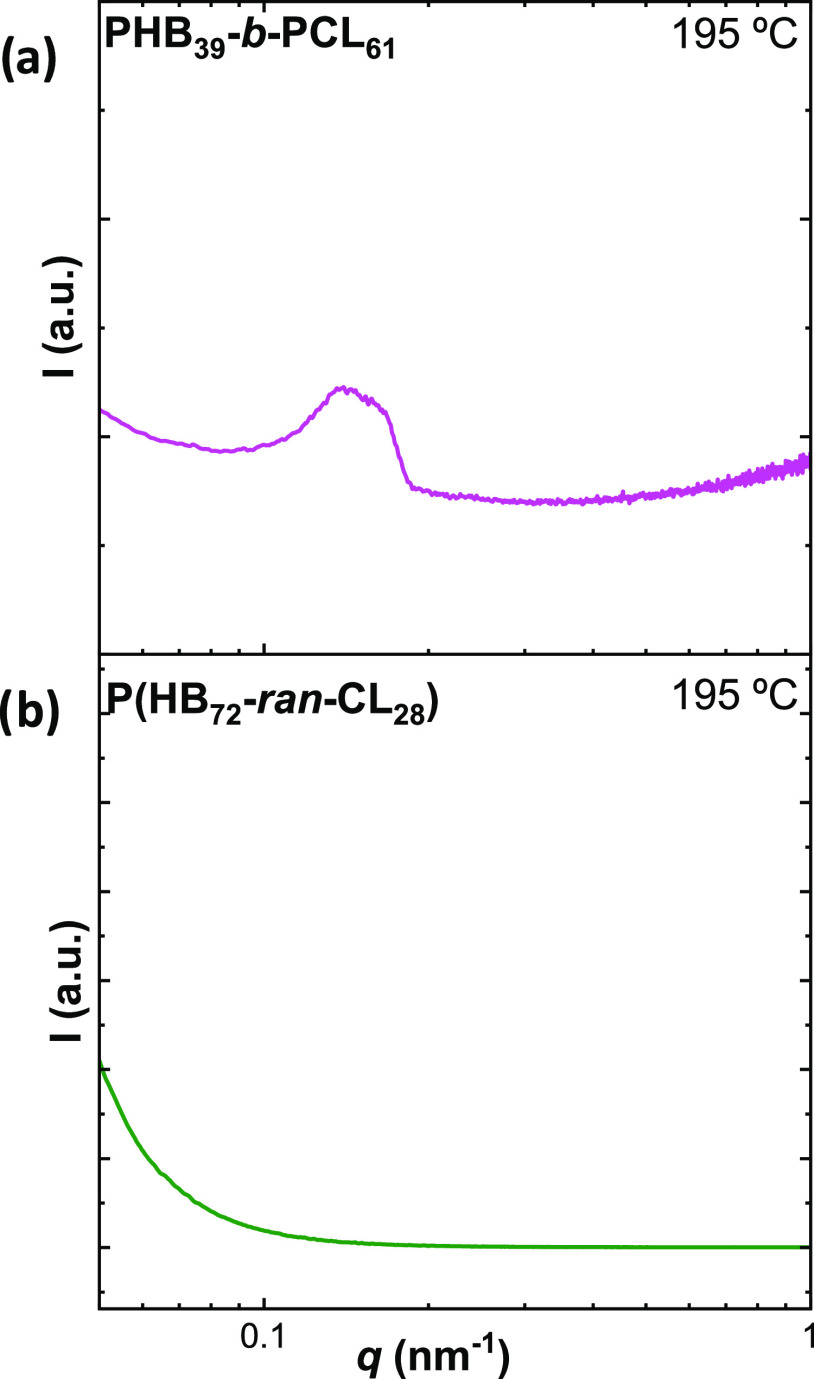
SAXS diffractograms acquired at 195 °C for PHB_39_-*b*-PCL_61_ (a) and P(HB_72_-*ran*-CL_28_) (b).

Figure 2 reports the plot of the intensity as a function of the scattering vector (*q*) for the PHB_39_-*b*-PCL_61_ (a) and P(HB_72_-*ran*-CL_28_) (b) samples in the melt. For the block copolymer, the presence of a diffraction peak at low *q* values is observed. This indicates that the blocks are segregated in the melt, as indicated by the calculation performed above, unlike the random copolymer, which obviously does not show phase separation, as the distribution of comonomers is random, and hence, it forms a single phase in the melt.

PHB and PCL have been reported to be immiscible in the melt in the case of blends.^38,58,59^ Furthermore, phase segregation in PHB-based materials has also been reported in the case of PHBV blends with high hydroxyvalerate (HV) content.^60−63^

In the case of the block copolymer, it is possible to calculate the value of *D* from the value of *q*_*max*_ according to the following equation
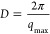
The resulting value is about 45 nm and can be attributed to the distance between the lamellae in a phase-segregated melt.

### 3.2 TGA and Nonisothermal DSC Results

Figure S3 shows the thermogravimetric curves of PHB and PCL neat samples reported in previous works^31,51^ and those of the PHB_39_-*b*-PCL_61_ and P(HB_72_-*ran*-CL_28_) copolymers. The homopolymers have a TGA curve consisting of a single degradation step, lower for PHB (about 280 °C) and higher for PCL (about 380 °C). The two copolymers exhibit two steps of degradation, as expected. Both in the block copolymer and in the random copolymer, the step at lower T can be attributed to the PHB component and the one at higher T to the PCL component.

**Figure 3:**
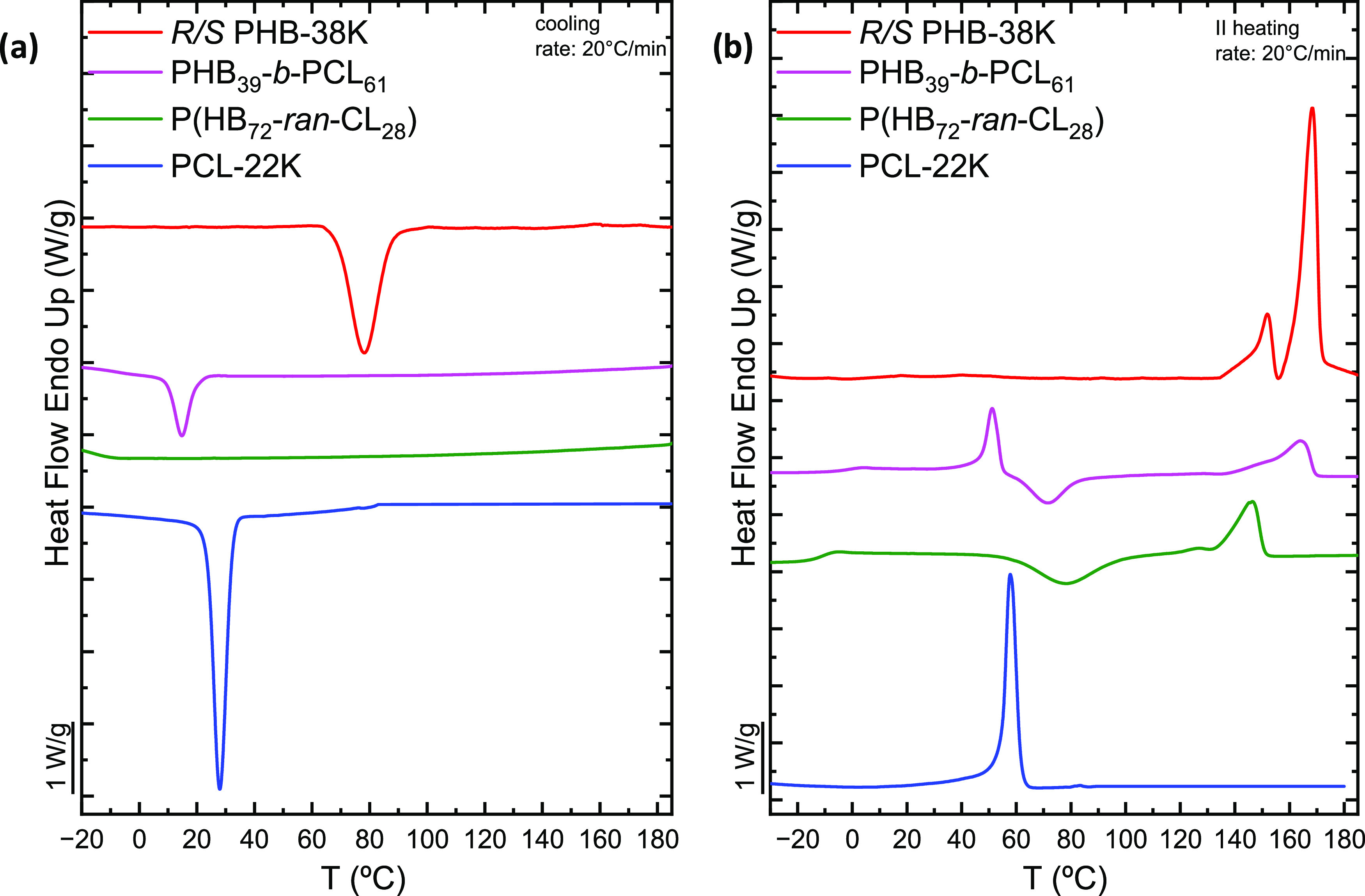
(a) DSC cooling scans at 20 °C/min and (b) subsequent DSC heating scans at 20 °C/min for PHB_39_-*b*-PCL_61_, P(HB_72_-*ran*-CL_28_), *R*/*S* PHB-38K, and PCL-22K.

Figure 3 reports the DSC cooling curves from melt (a) and the corresponding subsequent heating (b) of the samples involved in this study. The PHB_39_-*b*-PCL_61_ block copolymer exhibits a crystallization exotherm due to the crystallization of the PCL block, which, in the copolymer, crystallizes at lower temperatures than the previously studied homopolymer (blue curve,^51^). It should be noted that the PHB block cannot crystallize during cooling to 20 °C/min. In the heating scan (Figure 3b), the first melting endotherm that appears at lower temperatures (at approximately 51.1 °C) corresponds to the melting of PCL block crystals, which melt at lower temperatures than in the PCL homopolymer.

Immediately after the melting of the PCL block in Figure 3b, a cold-crystallization exotherm corresponding to the PHB block is observed at about 72 °C. Despite being absent in the reference neat PHB polymer (red curve in Figure 3b), this phenomenon has been reported for higher molecular weight PHB samples.^31^ This behavior can be attributed in part to the slightly higher molecular weight of the block copolymer’s PHB chains but primarily to the presence of the covalently bonded PCL block chains, which apparently reduce the crystallization capacity of the PHB block. During cooling from the melt at 20 °C/min, the PHB block was not able to crystallize, as opposed to the neat PBH employed here for comparison purposes. However, the PHB block can crystallize upon heating from the glassy state in the observed cold-crystallization exotherm. At higher temperatures (i.e., 164 °C), the melting peak of the PHB block is observed. The melting process is complex, and at higher magnification, a small cold-crystallization process is observed, as well as bimodal melting. The behavior somewhat resembles that of neat PHB, and it is typical of reorganization and recrystallization during the scan, as observed previously by us in this neat, chemically synthesized PBH material.^31^

During the cooling process, the P(HB_72_-*ran*-CL_28_) random copolymer does not crystallize, according to Figure 3a. In the following heating process, a phenomenon of cold crystallization followed by melting is observed. Given the temperatures at which these phenomena occur, they can be attributed to the PHB block chains, which cold crystallize and then melt. The amount of PCL within the random copolymer is too low to allow it to crystallize. But, precisely, the presence of randomly distributed PCL units in the copolymer lowers the melting point of PHB (and its crystallinity), as the PCL units interrupt the linear crystallizable sequences of PHB. The PHB phase in the PHB-*ran*-PCL copolymer has a melting peak at about 145 °C, lower than that of the block copolymer or neat PHB. As already observed in the case of the block copolymer and the neat polymer, the melting peak of PHB has a typical shape of crystal reorganization during heating.

Table 2 lists the thermal parameters obtained from the nonisothermal crystallization experiments, including the degree of crystallinity calculated as reported in Section 2.2.4. Considering the occurrence of the cold-crystallization phenomenon described above, two degrees of crystallinity are distinguished, one calculated at 25 °C and one calculated at 100 °C, during the melting process.

**Table 2:** Calorimetric Data Extracted from Figure 3 for *R*/S PHB-38K, PCL-22K PHB_39_-*b*-PCL_61_, and P(HB_72_-*ran*-CL_28_)

	*R*/S PHB-38K	PCL-22K	PHB_39_-*b*-PCL_61_	P(HB_72_-*ran*-CL_28_)
*T*_c/cc_ (°C)	78.0	28.0	14.8 (PCL block)	78.3
			71.4 (PHB block)	
Δ**H**_c/cc_ (J/g)	60	60	22 (PCL block)	37
			22(PHB block)	
*T*_m_ (°C)	151.8/168.5	58.9	51.2 (PCL block)	126.2/146.5
			164.0 (PHB block)	
Δ**H**_m_ (J/g)	14/87	60	21 (PCL block)	32
			27 (PHB block)	
*x*_c,25 °C_ (%)	41	43	25 (PCL)	0 (PHB)
			0 (PHB)	0 (PCL)
*x*_c,100 °C_ (%)	41	0	0 (PCL)	0 (PCL)
			46 (PHB)	22 (PHB)
*T*_g_ (°C)	1.4	–50.6	–3.9	–14.0
% PCL	0	100	61	28
*M*_n_ (kDa)	38	22	36 (Total)	75
			14 (PHB)	
			22 (PCL)	

It should be noted that at 25 °C, unlike the PHB homopolymer, the PHB component in the two copolymers has a degree of crystallinity equal to 0 since, in the cooling process, the chains did not crystallize. On the contrary, the PCL block is semicrystalline for both homopolymer and block copolymer cases.

At 100 °C, the degree of crystallinity of the PHB remains constant in the case of the homopolymer (i.e., 41%), while it reaches a value of 46% in the case of the block copolymer (similar to that of the neat PHB homopolymer, considering the error of the measurement, i.e., typically between 10 and 15%) and only 22% in the case of the random copolymer, as the PHB component cold crystallizes in the two copolymers during heating. The degree of crystallinity of the PCL component at 100 °C is equal to zero, as it is molten.

Summarizing, incorporating 28% PCL units randomly distributed within the PHB chains depresses its melting temperature by approximately 22 °C and reduces the nonisothermal crystallization substantially, as the material is not able to crystallize during cooling from the melt at 20 °C/min. Nevertheless, the PHB segments (72%) within the random copolymer can cold crystallize during heating (at 20 °C/min) to achieve a maximum degree of crystallinity that is only 22%, or about half the degree of crystallinity that neat PHB can develop during cooling from the melt. These results reveal the strong effects caused by random copolymerization with PCL. The PCL segments (28%) in the random copolymer are unable to crystallize (lowering even more the total crystallinity degree of the sample).

On the other hand, in the case of the block copolymer with 39% PHB, both blocks are able to crystallize, but the 61% PCL content also reduces the nonisothermal crystallization kinetics of the PHB block, and this component is not able to crystallize during cooling from the melt at 20 °C/min. The PHB blocks can only crystallize during heating from the glassy state (at 20 °C/min), but the crystals formed melt at slightly lower temperatures than those of neat PHB (i.e., 4.5 °C lower), while the degree of crystallinity of the PHB blocks is comparable within error to that of neat PHB.

### 3.3 Nonisothermal PLOM Results

The morphology of the samples was studied by PLOM to better understand the effect of phase segregation in the block copolymer and any differences between the block and random copolymer.

**Figure 4:**
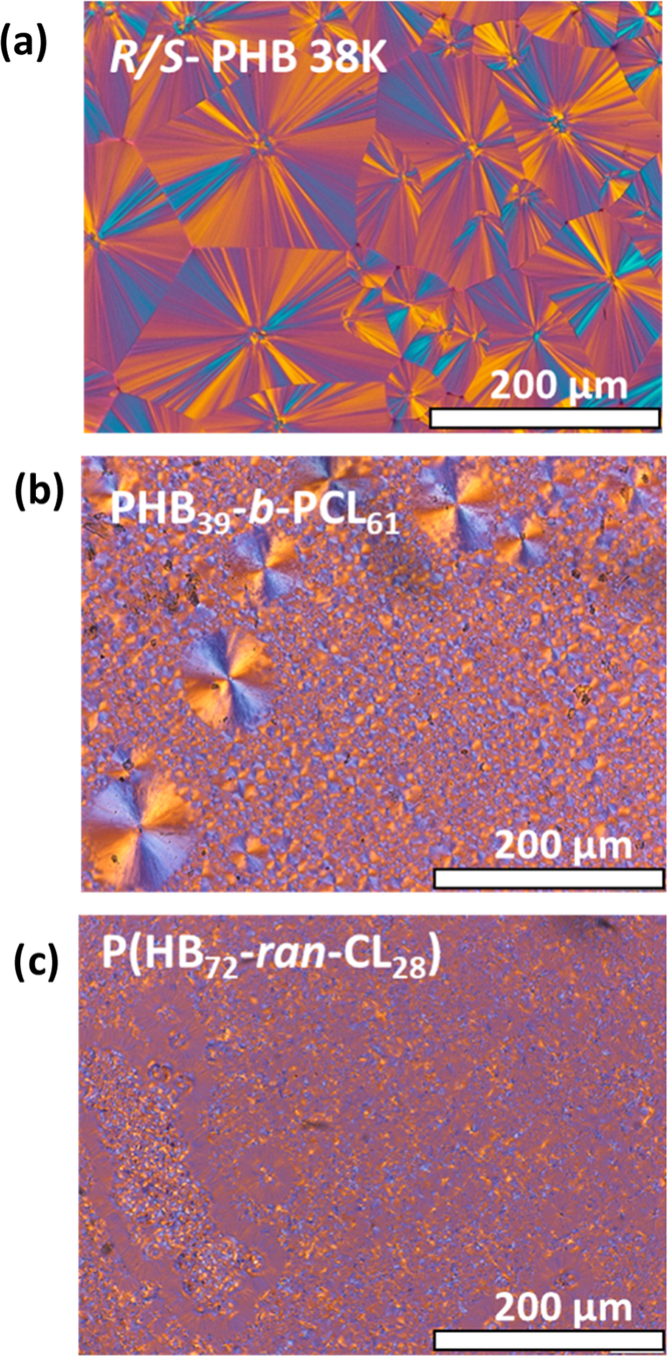
PLOM micrographs corresponding to the PHB superstructural morphology. For the neat *R*/S PHB-38K (a), the micrograph was taken after cooling it from the melt at 20 °C/min at 25 °C. In the case of the copolymer samples, the micrographs were taken during the second heating run at 100 °C for the PHB_39_-*b*-PCL_61_ sample (b) and at 107 °C for the P(HB_72_-*ran*-CL_28_) sample.

Figure 4 reports PLOM micrographs of the PHB_39_-*b*-PCL_61_ block and PHB_72_-*ran*-CL_28_ random copolymers compared with the PHB homopolymer. The micrograph in Figure 4a belongs to the homopolymer of PHB at room temperature taken after cooling from the melt at 20 °C/min. Instead, in the case of the block and random copolymers, the micrographs shown in Figure 4b,4c were obtained during the second heating process (immediately after cooling from the melt at 20 °C/min) at 100 °C for the block copolymer and at 107 °C for the random copolymer (both temperatures exceed the cold-crystallization temperature for the PHB component according to Figure 3b), since, as already observed previously by DSC (Figure 3a), no crystallization of the PHB component was detected in the copolymers during the cooling process from the melt.

The first aspect that can be noticed is the disparity in the nucleation density of PHB in the copolymer samples compared to neat PHB, which is characterized by a low nucleation density and, thus, large spherulitic sizes (Figure 4a). The nucleation density of the PHB component, as deduced by a large number of spherulites per unit area, is very high for the block copolymer PHB_39_-*b*-PCL_61_ (Figure 4b) and even higher in the random copolymer P(HB_72_-*ran*-CL_28_) (Figure 4c), compared with the PHB homopolymer (Figure 4a). In the case of the P(HB_72_-*ran*-CL_28_) random copolymer, Figure 4c is characterized by a very fine PHB microspherulitic morphology. In this copolymer, the PCL block does not crystallize, and even if it did, the micrograph was taken at temperatures well above the melting point of PCL crystals.

The formation of well-defined PHB spherulites in the PHB_39_-*b*-PCL_61_ block copolymer indicates that crystallization takes precedence over the phase segregation observed in the molten state detected by SAXS. This phenomenon occurs due to a breakout process during the heating DSC scan, triggered by the cold crystallization of PHB block chains. As can be seen from the micrograph shown in Figure 4b, in the PHB_39_-*b*-PCL_61_ block copolymer, the PHB block crystallizes, forming negative spherulites. This is clearly indicated by the first and third quadrant yellow extinction colors that can be seen when using a lambda red tint plate at 45° with respect to the polarizer direction (as we have done in this work^64^). This is a peculiar aspect since previous literature reports that PHB tends to form positive spherulites both in enantiomerically pure *R*-PHB of bacterial origin^55,65^ and in the case of synthetic origin PHB in the form of a racemic mixture *R*/*S*.^31^

This inversion in the sign of the spherulites has already been reported in the literature for PHB when it is blended with miscible polymers and, more specifically, in the case of blends with polymethyl acrylate (PMA)^66^ and polybutylene adipate (PBA).^67^ In the first case, a critical composition is reported at which the inversion occurs, i.e., 60% PHB and 40% PMA,^66^ and in the second case, the inversion is governed by the crystallization temperature, as low crystallization temperatures lead to the formation of negative spherulites in the PHB/PBA blend (50/50).^67^ In both cases, the inversion is due to the rotation of the lamellae with respect to the classical direction, which would make the spherulite positive. This optical sign switch is observable only when the PHB is in fully miscible systems with no phase separations or segregations. This could also be an explanation for the system studied in this paper, given the intermediate phase segregation that characterizes the PHB_39_-*b*-PCL_61_ sample (see Section 3.2).

### 3.4 Isothermal PLOM Results

As spherulites were detected in the PHB_39_-*b*-PCL_61_ block copolymer during the nonisothermal crystallization, isothermal crystallization experiments were conducted to evaluate spherulitic growth rates by PLOM. The sample was cooled rapidly from the melt (at a rate of 50 °C/min) to various isothermal crystallization temperatures ranging from 90 to 110 °C. The growth rate *G* (μm/s) of the spherulites was then determined by calculating the slope of the linear plot of spherulitic radius as a function of time for each crystallization temperature. Employing this approach, it was possible to follow the isothermal spherulitic growth from the melt specifically for the PHB block in the PHB_39_-*b*-PCL_61_ copolymer, as the crystallization temperatures for the PCL block are much lower. Unfortunately, attempts to follow the PCL block spherulitic growth failed, as the sample crystallized with a very high number of very small spherulites.

Typically, two phenomena^68,69^ compete in the trend of spherulitic growth rate as a function of temperature, which yields a bell-shaped curve. On the right side of the bell-shaped curve, as the temperature decreases, the growth rate increases. In this elevated temperature range (near the melting point), the growth rate is primarily influenced by secondary nucleation kinetics, which intensifies with supercooling until it reaches its peak level. At this maximum point, the melt viscosity has increased so much that diffusion takes over as the temperature is reduced. The rate at which crystals grow is controlled by the diffusion of polymer chains toward the crystallization front. As a result, the growth rate decreases with temperature. When a temperature value close to *T*_g_ is reached, the growth rate decreases gradually until it reaches a value of zero, as long-range chain mobility stops below *T*_g_.

**Figure 5:**
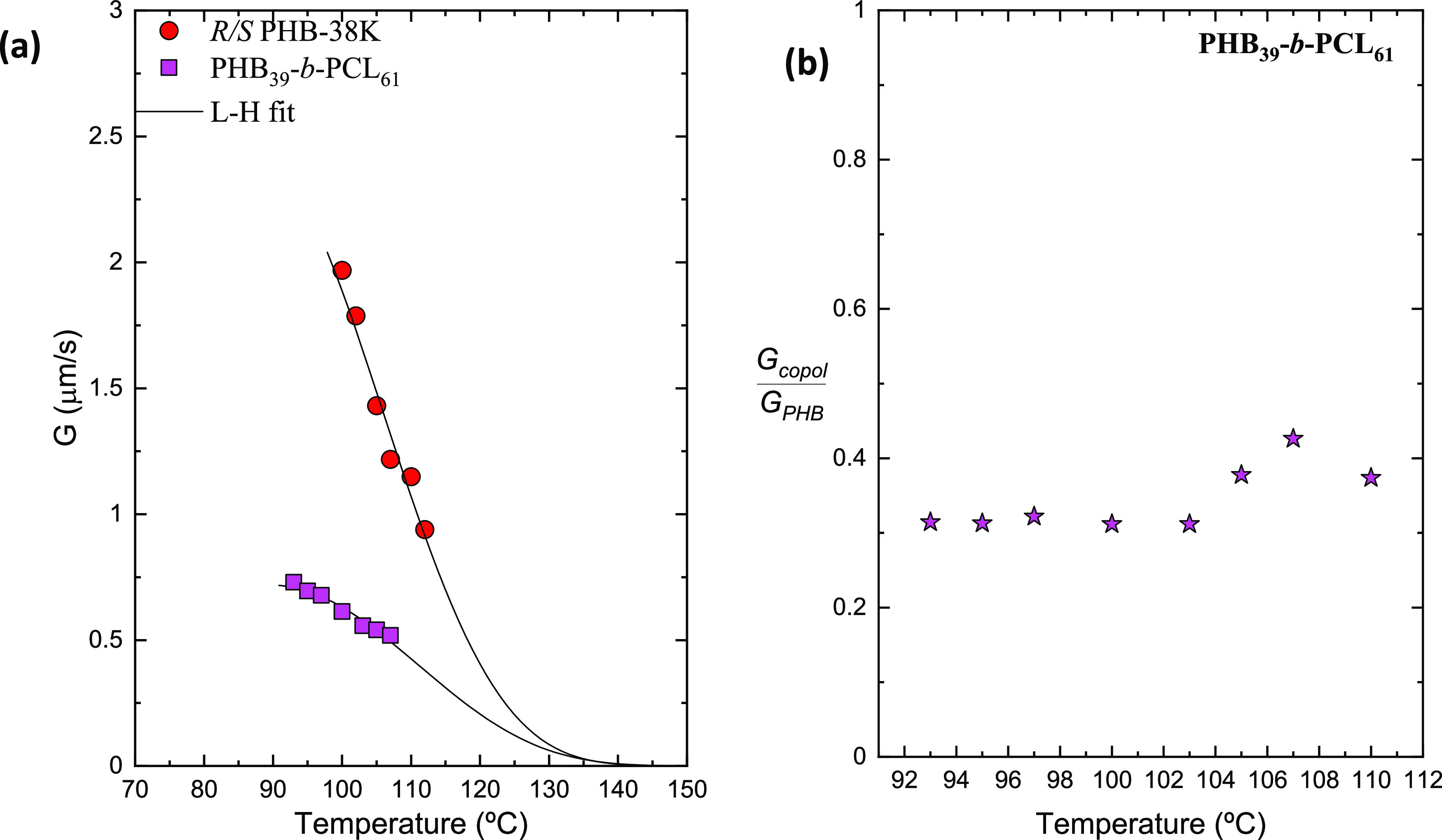
Spherulitic growth rate (*G*) as a function crystallization temperature (a) for the PHB_39_-*b*-PCL_61_ sample compared with *R*/*S* PHB-38K,^31^ and the normalized spherulitic growth rate  over crystallization temperature (b). The solid lines in the graph on the left are fits to the Lauritzen and Hoffman equation.

In Figure 5a, the results of the spherulitic growth rate as a function of *T*_c_ are reported. For the PHB_39_-*b*-PCL_61_ block copolymer, it was possible to measure the growth rate of the PHB block only on the right side of the typical bell-shaped curve (magenta squares in the graph), as after rapid cooling to crystallization temperatures below 90 °C, the sample isothermally crystallized into many small spherulites (due to a high nucleation density), which saturated the observation area. The spherulitic growth rates for the reference PHB sample have been reported in a previous work^31^ and are included in Figure 5a for comparison purposes (red dots in the graph).

In both samples, secondary nucleation dominates the superstructural growth and determines the trend of the graph in Figure 5a. The *T*_c_ range is similar for both samples, and it is evident that the PHB block in the copolymer crystallizes more slowly than the reference pure PHB. Note that the number average molecular weight of the reference material is 38.000 g/mol, while that of the PHB block is only 14.000 g/mol. One would expect lower molecular weight PHB homopolymer chains to crystallize faster.^25^ However, in this case, the 14.000 g/mol PHB chains are covalently bonded to PCL chains, and this seems to be the determining factor in the observed behavior. In fact, the *G* values corresponding to the PHB block within the PHB-*b*-PCL copolymer are always lower than those of the reference neat PHB. The reason is probably due to the presence of the covalently bonded PCL block chains, which are molten at the crystallization temperature of the PHB block, and their high mobility interferes with the spherulitic growth of the PHB block chains at the growth front, slowing down the crystal growth of the PHB block. This has also been reported for samples of the PLLA-*b*-PCL block copolymer in which the molten PCL block chains slow down the crystallization of the PLLA block^70^ in view of their weakly/intermediate segregated strength in the melt, such as the system under study.

Figure 5b shows the *G* value of the PHB_39_-*b*-PCL_61_ copolymer divided by the *G* value of neat PHB as a function of temperature: it can be noticed that in the entire *T*_c_ range, the normalized *G* value is, on average, 0.35 and this indicates that the PHB_39_-*b*-PCL_61_ block copolymer has a 65% slower spherulitic growth rate than the PHB homopolymer sample employed here for comparison purposes.

**Figure 6:**
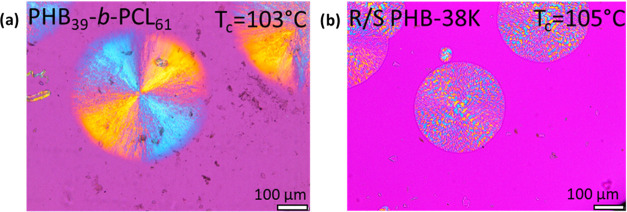
PLOM micrographs taken at the indicated *T*_c_ for PHB_39_-*b*-PCL_61_ (a) and *R*/*S* PHB-38K^31^ (b).

Figure 6 shows two PLOM images taken at the indicated *T*_c_ values for PHB_39_-*b*-PCL_61_ (a) and *R*/*S* PHB-38K^31^ (b). The difference in morphology is evident, as the presence of negative spherulites is observed in the PHB block spherulites, contrary to what is observed in the reference PHB, which has an average positive sign. Furthermore, the reference PHB is characterized by banded spherulites (Figure 6b), unlike the PHB block within the copolymer, which forms very clear Maltese crosses without any banding.

The theory of Lauritzen and Hoffman^71^ was used to fit the experimental data, and the solid lines in Figure 5a are the result of this fit. The parameters obtained from the fits are shown in the SI, as well as a description of the equation employed. Even though the theory fits the data points, as shown in Figure 5a, the fitting parameters have values that are very close to and probably within the errors involved in the fits. For the random P(HB_72_-*ran*-CL_28_) copolymer, it was not possible to follow the spherulite growth of the PHB component, as the spherulites are too small (even at high *T*_c_ values) and saturate the observation area very quickly as a result of a nucleation density higher than that observed for the spherulites of the PHB block within the block copolymer.

### 3.5 Study of the Overall Crystallization Kinetics by DSC

DSC was used to conduct isothermal crystallization experiments aimed at investigating the overall crystallization kinetics resulting from the combined effects of primary nucleation and the growth of superstructural aggregates. The discussion below is divided into two sections for ease of understanding. Indeed, in Section 3.5.1, the crystallization of the PHB block in the PHB_39_-*b*-PCL_61_ and P(HB_72_-*ran*-CL_28_) copolymers is discussed in comparison with the neat PHB. In Section 3.5.2, the crystallization of the PCL block in the PHB-*b*-PCL block copolymer is presented for the case in which the PHB block was quenched to the amorphous state and also for the different cases in which it was allowed to crystallize first.

One of the ways to analyze the overall crystallization kinetics results is to fit them with the Avrami theory^72−74^ represented by the following equation:
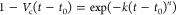
in which *V*_c_ is the relative transformed fraction by volume into the semicrystalline state, *t is* the experimental time, *t*_0_ is the induction time, *k* represents the overall crystallization rate constant, and *n* is the Avrami index, which is connected to the nucleation rate and the growth dimensionality of the crystals.

#### 3.5.1 PHB Block Crystallization within PHB_39_-*b*-PCL_61_ and Crystallization of the PHB Component within P(HB_72_-*ran*-CL_28_)

**Figure 7:**
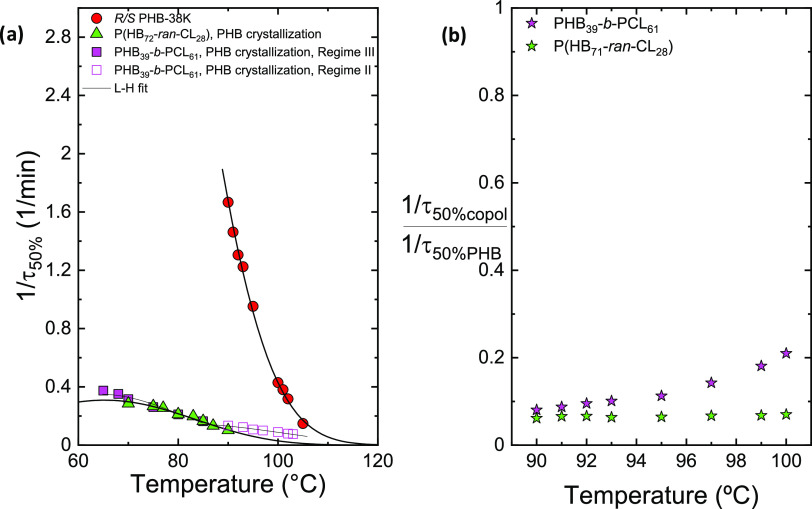
Inverse of half-crystallization time (1/τ_50%_) (a) and normalized inverse of half-crystallization time (1/τ_50%_) (b) as a function of *T*_c_ for PHB block crystallization in PHB_39_-*b*-PCL_61_ and P(HB_72_-*ran*-CL_28_) samples in comparison with *R*/*S* PHB-38K.^31^ The solid lines in (a) represent the fits to the Lauritzen and Hoffman theory.

As previously mentioned, the results of the global isothermal crystallization of the PHB component in the PHB_39_-*b*-PCL_61_ and P(HB_72_-*ran*-CL_28_) samples are reported in this section. Figure 7a reports the inverse half-crystallization time, 1/τ_50%_, versus the crystallization temperature for the PHB component in the PHB_39_-*b*-PCL_61_ and P(HB_72_-*ran*-CL_28_) copolymers and in the reference PHB. The value of 1/τ_50%_ is the inverse of the time that the polymeric materials need during an isotherm to crystallize to 50% of their relative crystallinity. Experimentally, this parameter contains two contributions, namely, nucleation and superstructural growth; in fact, it is an experimental measure of the overall crystallization rate.

As depicted in Figure 7a, the PHB component in the PHB_39_-*b*-PCL_61_ and P(HB_72_-*ran*-CL_28_) samples crystallizes more slowly than in the reference neat PHB. The reason for this behavior may be attributed to the presence of PCL, which, at the crystallization temperatures of PHB, is in the molten state and interferes with the PHB component crystal growth, as argued above for the block copolymer case, resulting in a decrease in the overall crystallization as well. For the random copolymer, a plasticization effect could be expected, as the *T*_g_ of the copolymer is lower than the *T*_g_ of neat PHB, as predicted for a random copolymer, see Table 2. This behavior was also found in the case of random copolymers composed of PBS and PCL, in which it was also observed that a solvent-type effect increased as the amount of PCL increased, slowing down the crystallization rate of PBS.^75^

The reduction of the overall crystallization rate of the copolymers compared to that of the neat reference polymers has been thoroughly investigated in the existing literature. One example was reported by Arandia et al.,^76^ in the case of random copolymers based on polybutylene succinate and polybutylene azelate: the incorporation of units of BAz results in an increase in the density of nuclei but a decrease in the overall crystallization rate of PBS. A similar situation arises for many random copolyesters, as reported in ref^77^

The solid lines in Figure 7a correspond to fits with the Lauritzen and Hoffman equation. As can be seen, in the case of PHB_39_-*b*-PCL_61_, the fit was performed with two crystallization Regimes: for low *T*_c_, the fit was performed with Regime III, and for high *T*_c_ with Regime II. According to the L–H theory, three Regimes are distinguished for the description of two competing phenomena, which are the creation of new nuclei and the deposition of chains on the lateral surface of the nuclei to complete their growth. In Regime I, the secondary nucleation rate is extremely reduced; in Regime II, the secondary nucleation rate and lateral growth rates are comparable; and in Regime III, the secondary nucleation rate is the fastest. This behavior was not found in the reference neat PHB nor in the P(HB_72_-*ran*-CL_28_), in which the fits were performed with only one Regime (i.e., Regime II). The presence of these two Regimes, in the block copolymer case, is due once again to the molten PCL, which interferes with the crystallization of the PHB block and is also found in the case of polypropylene/poly(ethylene-octene)^78^ blends and polyethylene(butylene/diethylene succinate) block copolymers.^79^ Regarding the P(HB_72_-*ran*-CL_28_) random copolymer, PCL is present in very small quantities compared to PHB, and this probably does not interfere with the Regime of crystallization of PHB, which takes place in Regime III only. The results of the Lauritzen and Hoffman fit are listed in Table S2, where the correct relationship between *K*_*g*_^τ^ (II) and *K*_*g*_^τ^ (III) is observed, which is around 2 for block copolymer PHB_39_-*b*-PCL_61_.

Figure 7b shows the value of 1/τ_50%_ of PHB_39_-*b*-PCL_61_ and P(HB_72_-*ran*-CL_28_) copolymers divided by the value of 1/τ_50%_ of neat PHB with respect to the crystallization temperature. It can be noticed that in the case of the random copolymer, this value is almost constant and always less than 0.1. In the case of the block copolymer, there is an increase in the normalized value of 1/τ_50%_, which is approximately 0.2 in the case of high *T*_c_. If we compare Figure 5b with Figure 7b, it can be observed that the reduction in the spherulitic growth rate leads to a normalized ratio of the growth rate of 0.3, which increases to 0.4 at high temperatures. These values are higher than the ratios of normalized overall crystallization rates (0.1–0.2), and this indicates that the decrease in the overall crystallization rate of the PHB component within the copolymers is influenced significantly by both nucleation and spherulitic growth rate.

Both the PHB components in the block copolymer and the random copolymer crystallize more slowly than the reference PHB homopolymer (90% slower in the random copolymer and 80% slower in the block copolymer). Considering Figure 5a, where it was not possible to measure the spherulitic growth of the random copolymer due to its high nucleation density (see Figure 4c), it can be considered that the slow and determining step for the overall crystallization rate is the spherulitic growth rate. In spite of the fact that nucleation is enhanced in the PHB component of the copolymers, their overall crystallization is much smaller than that in neat PHB because of the slow spherulitic growth.

**Figure 8:**
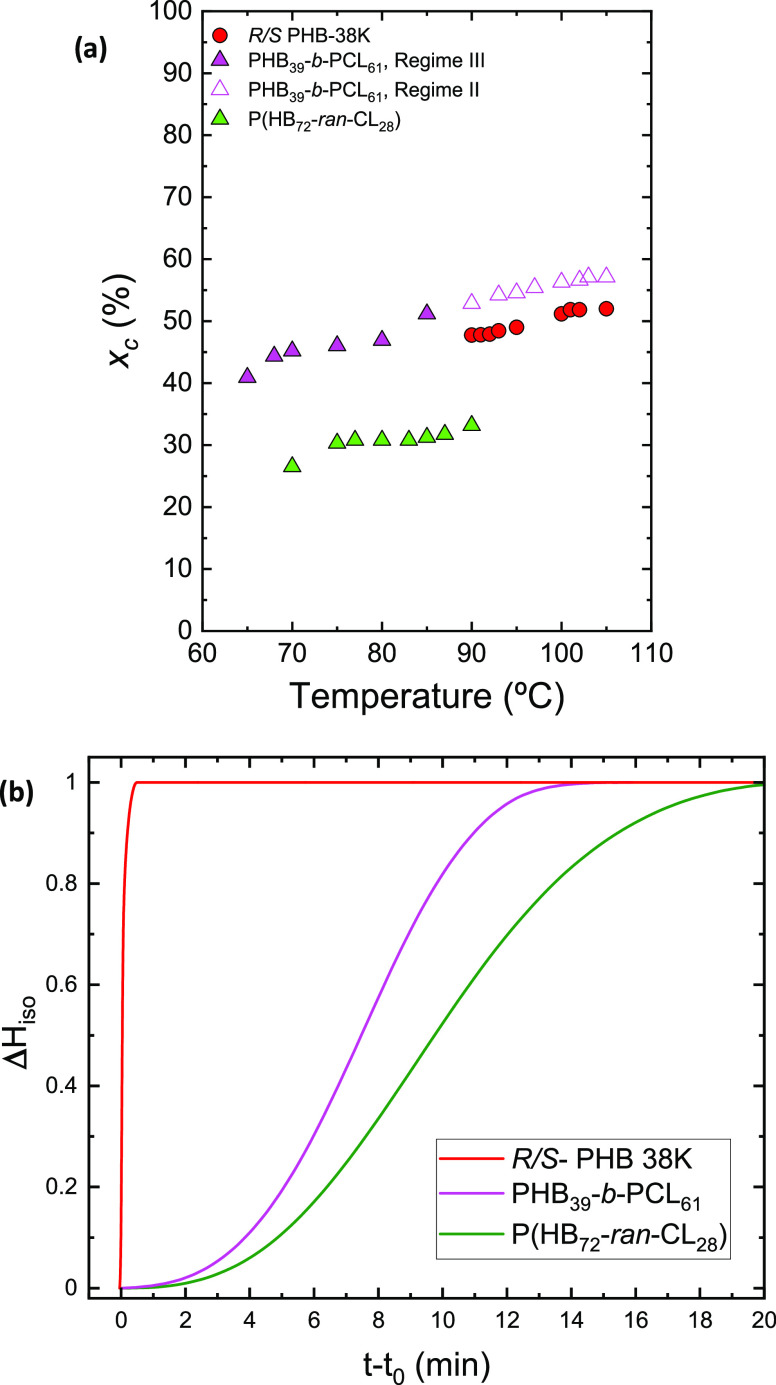
Degree of crystallinity (*x*_c_) obtained during isothermal crystallization as a function of *T*_c_ (a) and enthalpy obtained during the isothermal crystallization process (Δ*H*_iso_) at *T*_*c*_ = 90 °C as a function of time (b).

Figure 8a reports the degree of crystallinity (*x*_c_) obtained at the end of the isothermal crystallization process for the two copolymers and the reference PHB as a function of the crystallization temperature. The value of the degree of crystallinity increases with the increase of the crystallization temperature for all of the samples, and in the case of the block copolymer, it reaches values that are very similar to those of the reference PHB, while in the case of the random copolymer, the values are significantly lower as expected. In the random copolymer, the PHB chains are interrupted by randomly placed units of PCL, which limit the maximum degree of crystallinity achieved. It is important to realize that even though the crystallinity degree achieved at the end of the crystallization period is the same for the PHB block and neat PHB, their crystallization kinetics are very different (as indicated in Figure 7). Therefore, the achievement of this similar degree of crystallization upon saturation can only be achieved at extremely different times at the same crystallization temperatures.

Figure 8b shows the progression of the crystallization enthalpy obtained during the isothermal crystallization process at *T*_c_ = 90 °C for the *R*/*S* PHB-38K, PHB_39_-*b*-PCL_61_, and P(HB_72_-*ran*-CL_28_) samples as a function of time. It is observed that the time required for the material to crystallize completely is very small (less than 0.5 min) in the case of the reference PHB homopolymer compared to that in the block and random copolymer. This corroborates the overall crystallization findings obtained from isothermal crystallization experiments and is shown in Figure 7. It should also be noted that the crystallization enthalpy in Figure 8b is normalized by dividing it by the maximum enthalpy achieved after crystallization has saturated, but if Figure 7a is observed, it can be realized that in the case of the random copolymer, not only does the PHB component crystallize much slower than neat PHB but also it achieves a final degree of crystallinity which is substantially lower.

Copolymerizing PHB with PCL provokes higher nucleation, which is normally related to better optical properties (higher transparency) and also a lower degree of crystallinity, at least in the random copolymer case (or in the block copolymer case, depending on the cooling rate or crystallization time). Lower degrees of crystallinity in PHB with smaller spherulites can produce much tougher materials from a mechanical point of view than brittle neat PHB.^32^

The experimental data of isothermal crystallization have been fitted, as described previously, with the Avrami theory, and the description of the results is reported in the SI.

#### 3.5.2 PCL Block Crystallization in PHB_39_-*b*-PCL_61_ from Crystalline and Amorphous PHB

This section reports the results of the isothermal crystallization of the PCL block in the PHB_39_-*b*-PCL_61_ sample, compared with the results obtained for a reference neat PCL.^51^ It should be observed that the isothermal crystallization of PCL was performed by using two different pathways. In the first, the sample rapidly cooled from the molten state at a rate of 60 °C/min directly to the crystallization temperature of PCL, and under these conditions, the PHB block was not capable of crystallizing, as was demonstrated by subsequent heating runs after the PCL block crystallization (where the PHB cold crystallized and melted with identical enthalpies). Therefore, the block of PCL was crystallized isothermally, as the PHB block was kept amorphous. In the second thermal protocol, the PHB_39_-*b*-PCL_61_ sample was first cooled from the melt as in Figure 1 (at 20 °C/min), then heated until 100 °C to allow for the PHB cold crystallization at 20 °C/min. Then, the samples were quenched at 60 °C/min to the isothermal crystallization temperature to measure the heat evolved as a function of time in the DSC corresponding to the PCL block in the presence of the PHB block crystals. After the isothermal step was completed, the sample was heated to 100 °C (at 20 °C/min) to melt only the PCL crystals and then quenched again at 60 °C/min to the next chosen *T*_c_ value.

**Figure 9:**
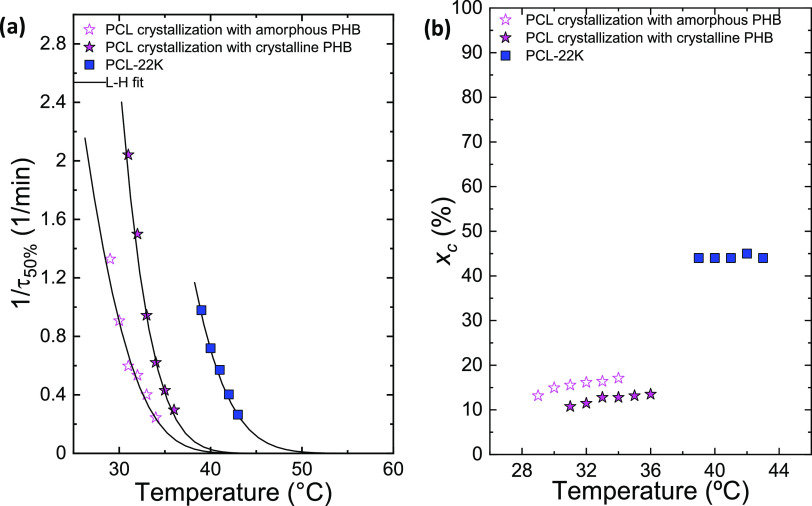
Inverse of half-crystallization time (1/τ_50%_) (a) and degree of crystallinity (*x*_c_) calculated during isothermal crystallization as a function of *T*_c_ for PCL block crystallization from crystalline PHB, full stars, and amorphous PHB, empty stars, in PHB_39_-*b*-PCL_61_ sample in comparison with PCL-22K.^51^ The solid lines in (a) represent the fits to the Lauritzen and Hoffman theory.

In Figure 9a, the inverse of the half-crystallization rate (1/τ_50%_) is reported over *T*_c_. Both in the case of amorphous and semicrystalline PHB block, the crystallization of the PCL block is always slower than neat PCL, but a nucleating effect of the PHB block crystals on the PCL block can be observed when the PHB block is in the semicrystalline state. Thus, the PCL block crystallization is always faster when the PHB block is semicrystalline for the same *T*_c_ value. This fact indicates that PHB crystals act as nucleating agents for PCL chains, which, in this way, crystallize more rapidly than when the PHB block is in an amorphous state.

Figure 9b shows the degree of crystallinity (*x*_c_) calculated at the completion of the isothermal crystallization process for the PCL block, in the case of amorphous and semicrystalline PHB block, and for the reference PCL. For neat PCL, the degree of crystallinity is constant with temperature. However, in the case of the PCL block, there is a small increase in the crystallinity degree with *T*_c_. It should be noticed that the crystallinity degree of the PCL block is between 10 and 15%, while that of neat PCL reaches 45%. In the case where the PHB block is semicrystalline, the degree of crystallinity is even smaller than that of the amorphous PHB block. The effect of the PHB block, therefore, decreases not only the crystallization rate of the PCL but also the amount of crystallinity it can achieve. When the PHB block is allowed to crystallize first, at high temperatures, the spherulites formed are templates for the crystallization of the PCL block. As the PCL block is covalently bonded, when the PHB block chains crystallize, the PCL block chains are segregated to the interlamellar amorphous regions between crystalline PHB lamellae. Subsequently, when the material is further cooled from its molten state, the PCL can solely crystallize within the limited spaces present between the crystalline PHB lamellae. A similar situation occurs in many double crystalline block copolymers, as reviewed elsewhere. So, it is not surprising that the PCL block crystallizes at the slowest rate when it does so inside the previously crystallized PHB spherulites, generating double crystalline spherulites. It is more surprising that quenching the sample to prevent the PHB block chains from crystallizing also causes such an important retardation in the PCL crystallization. This behavior may be due to the intermediate segregation strength present in this block copolymer.

The results of fitting the L–H and Avrami theories to the crystallization kinetics of the PCL block are presented in the SI.

## 4 Conclusions

We have studied how the inclusion of PCL units in a random or blocky arrangement influences the morphology, thermal properties, and crystallization kinetics of PHB. The PHB_39_-*b*-PCL_61_ block copolymer exhibited an intermediate segregation strength in the melt, while the P(HB_72_-*ran*-CL_28_) random copolymer showed the expected single-phase melt. Nevertheless, the crystallization of the PHB block “breaks-out” from the phase-segregated structure of the melt to form well-developed negative spherulites. This is a novel finding, as PHB normally forms positive spherulites; therefore, the covalently bonded PCL block can alter the optical properties of the PHB block spherulites.

Neat PHB exhibits a low nucleation density, resulting in the formation of large spherulites that concentrate stresses and are mostly responsible for its characteristic brittleness, together with its high degree of crystallinity. The block copolymer sample exhibits a higher nucleation density and smaller spherulites, on average. However, the random copolymer displayed an extremely fine microspherulitic texture that would be highly beneficial for both mechanical properties and transparency. In addition, both block and random copolymer samples examined here presented a much lower spherulitic growth rate and overall crystallization rate. In the case of the block copolymer, the PHB block is capable of developing a degree of crystallinity comparable to that of neat PHB but at much higher crystallization times while being covalently bonded to a softer PCL block with reduced crystallinity degree. The PHB component of the random copolymer displays a much lower *T*_m_ value and crystallinity degree than neat PHB, and the PCL component does not crystallize as it is a minor component randomly distributed along the chains. Therefore, this random copolymer is an attractive biodegradable material with improved processing (due to its lower melting temperature) and potentially much better mechanical and optical properties than neat PHB in view of its lower degree of crystallinity and microspherulitic morphology.

In the special scenario of the PHB_39_-*b*-PCL_61_ diblock copolymer, both blocks can crystallize, and we demonstrated that if the PHB is crystallized first at higher temperatures, it can nucleate the PCL block. However, if the PHB block is quenched so that it remains amorphous during the crystallization of the PCL block, the isothermal crystallization kinetics is faster than when the PHB block is semicrystalline but still much lower than neat PCL. In both cases, the degree of crystallinity attained during isothermal crystallization by the PCL block is much lower (between 10 and 20%) than in the case of neat PCL with comparable chain length (which can reach approximately 40% crystallinity during isothermal crystallization).

## Supporting Information Available

Reactions and details of the synthesis of the copolymers studied in this work (Figure S1); ^1^H NMR spectra (Figure S2); TGA results (Figure S3); parameters obtained from isothermal crystallization employing PLOM (Table S1); parameters obtained from isothermal crystallization employing DSC (Table S2); examples of Avrami fits (Figures S4–S7); parameters obtained from the Avrami fits (Tables S3–S6) (PDF)
