# Supplementary material for: Tailoring the Nucleation and Crystallization Rate of Polyhydroxybutyrate by Copolymerization
Source: Biomacromolecules. 2023 Oct 2;24(11):5328–41. doi: 10.1021/acs.biomac.3c00808 (PMC10646943; doi:10.1021/acs.biomac.3c00808)
Supplement: Supplementary file 1 — bm3c00808_si_001.pdf [file bm3c00808_si_001.pdf]

Supporting Information

Tailoring the nucleation and crystallization rate of Polyhydroxybutyrate by copolymerization

Maria Rosaria Caputo[a], Changxia Shi[b]. Xiaoyan Tang[b], Haritz Sardon*[a], Eugene Y.-X. Chen*[b] Alejandro J. Müller*[a, c]

[a] POLYMAT and Department of Polymers and Advanced Materials: Physics, Chemistry and Technology, Faculty of Chemistry, University of the Basque Country UPV/EHU, Paseo Manuel de Lardizabal 3, 20018 Donostia-San Sebastián, Spain

[b]Department of Chemistry, Colorado State University, Fort Collins, Colorado, 80523-1872, United States

[c] IKERBASQUE, Basque Foundation for Science, Plaza Euskadi 5, 48009 Bilbao, Spain

*Corresponding authors: haritz.sardon@ehu.eus; eugene.chen@colostate.edu; alejandrojesus.muller@ehu.es

Details of the synthesis carried out by Tang *et al*., [1]

1. Block copolymer PHB_{39}-*b*-PCL_{61}
rac-DL^{Me} = 4.132 g (24 mmol), ε-CL = 1.370 g (12 mmol), Cat **2** (La-trityl) = 51 mg (45 µmol), BnOH = 4.87 mg (45 µmol), DCM = 36 mL, time = 24 h
(Conditions: *rac*-DL^{Me}/ ε-CL = 2/1, [*rac*-DL^{Me} + ε-CL]/[**2**]/[BnOH] = 800/1/1, 24 h)

2. Random copolymer P(HB_{72}-*ran*-CL_{28})
rac-DL^{Me} = 5.165 g (30 mmol), ε-CL = 34.242 g (300 mmol), Cat **1** (Y-trityl) = 162 mg (150 µmol), BnOH = 16.2 mg (150 µmol), DCM = 30 mL, time = 25 min
(Conditions: *rac*-DL^{Me}/ ε-CL = 1/10, [*rac*-DL^{Me}]/[**1**]/[BnOH] = 200/1/1, 25 min)

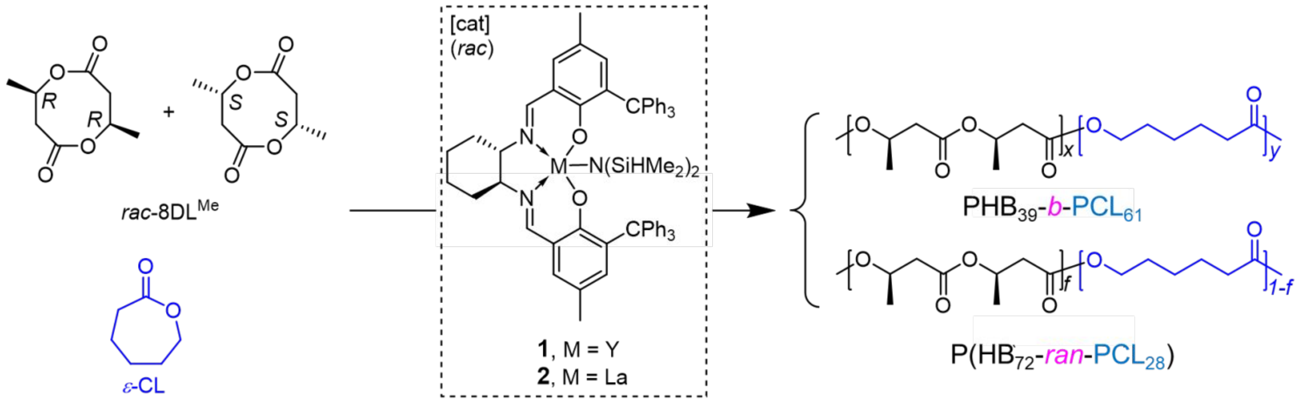

Figure S1. Scheme of reactions

a)

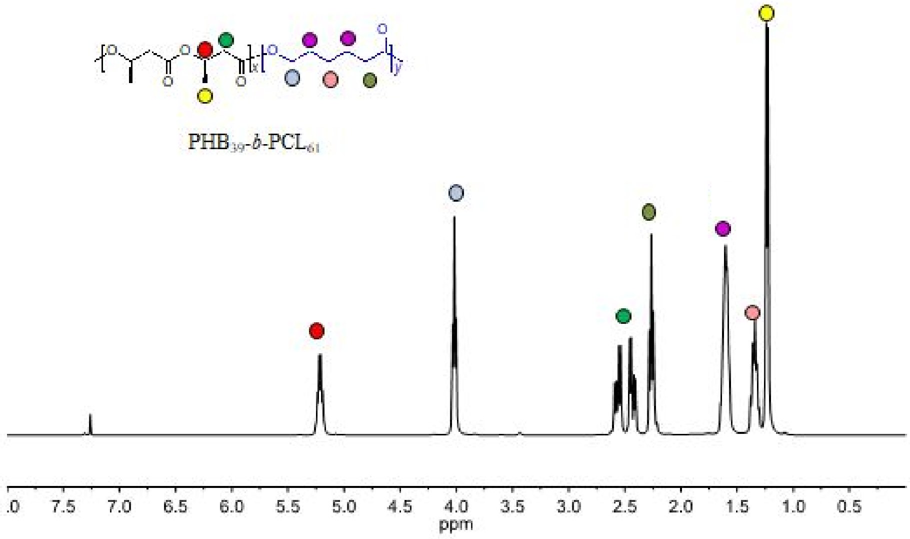

b)

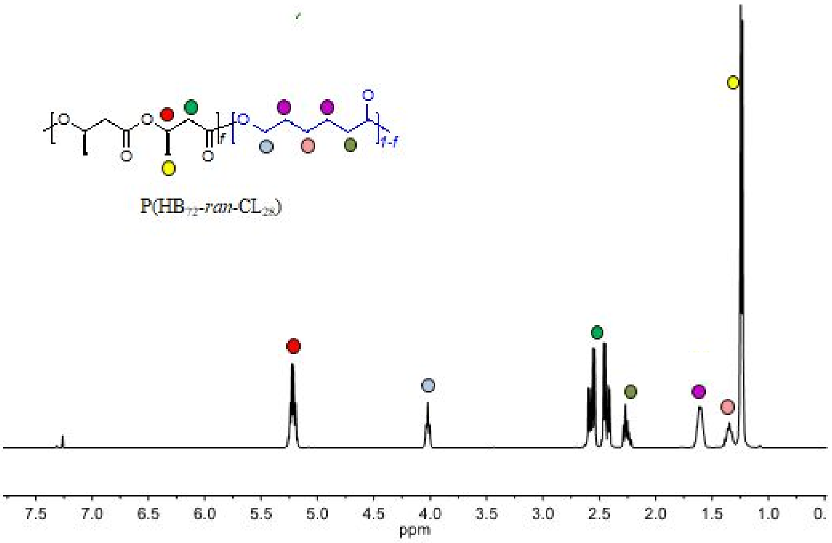

Figure S2. . ^{1}H NMR spectroscopy in $CDCl_3$ of PHB_{39}-*b*-PCL_{61} (a) and P(HB_{72}-*ran*-CL_{28}) (b)

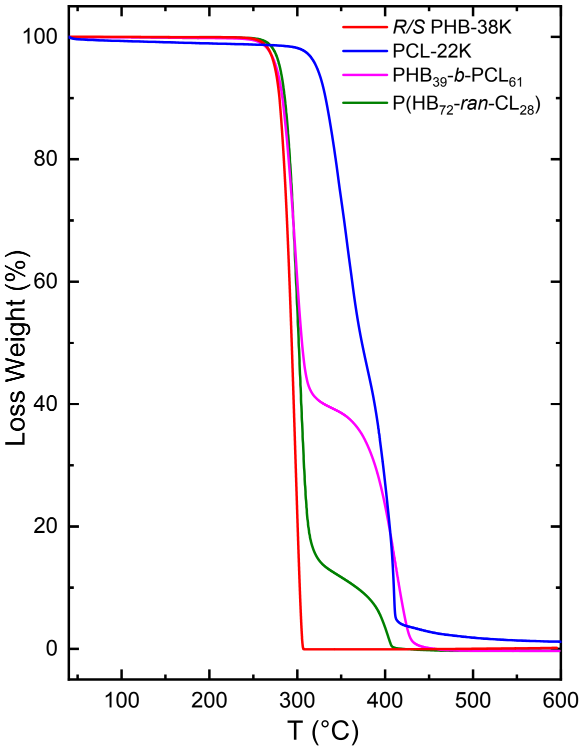

Figure S3. Weight loss (%)as a function of the temperature (°C) for PHB_{39}-*b*-PCL_{61}, P(HB_{72}-*ran*-CL_{28}), *R/S* PHB-38K and PCL-22K.

Table S1. Isothermal kinetics data parameters described in Paragraph 3.4 derived from experimental results obtained by PLOM. The data for *R/S* PHB-38K are present in the literature[2].

Sample	G^0(cm/s)	K^G_g (K^2)	σ (erg/cm^2)	σ_e (erg/cm^2)	q (erg)	R^2
PHB_{39}-*b*-PCL_{61}	3.85	$3.67x10^5$	8.39	228.2	$8.42x10^{-13}$	0.995
R/S PHB-38K[2]	136	$3.33x10^5$	8.39	284.2	$1.05x10^{-12}$	0.986

The used Lauritzen-Hoffman equation[3] was the following:

$$G = G_o\, exp\left[\frac{-U^*}{R(T_c - T_0)}\right]\left[\frac{-K_g^G}{fT(T_m^0 - T_c)}\right]$$

In this equation G_o is a constant that includes all terms that are not temperature dependent, U^* is an energy that in this study, assumes the value of 1550 cal/mol. More specifically it is a transport activation energy for the diffusion of polymer chains; R is the universal gas constant, T_c is the crystallization temperature, T_0 is 30ºC degrees lower than the T_g and is the temperature that corresponds to the frozen movement of the chains, T_m^0 is the equilibrium melting temperature and f is a temperature correction factor represented by the following expression $2T_c/(T_m^0 + T_c)$. Furthermore, K_g^G is a constant proportional to the energy barrier for the spherulitic growth or secondary nucleation, given by the expression:

$$K_g^G = \frac{j\, b_0\, \sigma\sigma_e T_m^0}{k\, \Delta H_m^0}$$

j assumes the value of 2 for the Regime II crystallization, a regime where the secondary nucleation and the spread of the nucleus on the growth front are equivalent[4], b_0 is the chain's width, σ represents the lateral surface free energy, σ_e is the fold surface energy, k is the Boltzmann constant and is the equilibrium latent heat of fusion. If ln $G + \frac{-U}{R\,(T_c - T_0)}$ is plotted versus$\frac{1}{T_c(\Delta T)f}$, it is possible to obtain a straight line where K_g^G is the slope and G_o the intercept. Through K_g^G, it could be possible to calculate the $\sigma\sigma_e$ value, and by the expression $\sigma = 0.1\Delta H_m^0\sqrt{a_0 b_0}$, where $a_0 b_0$ is the chain cross-sectional area, it is possible to obtain the values of σ and σ_e. Furthermore, it is also possible to calculate q, the work that macromolecules do to fold as $2a_0 b_0 \sigma_e$. [5]

Table S2. Overall isothermal kinetics data parameters described in the Paragraph 3.5 derived from experimental results obtained by DSC.

**Sample**	$1/\tau_0$**(1/s)**	K^{τ}_{g} (K^2)	σ **(erg/cm²)**	σ_e **(erg/cm²)**	q **(erg)**	R^2
PHB_{39}-*b*-PCL_{61} Regime III (PHB crystallization)	7.40×10^{10}	7.79×10^{5}	8.39	332.5	1.23×10^{-12}	0.994
PHB_{39}-*b*-PCL_{61} Regime II (PHB crystallization)	1.90×10^{5}	3.74×10^{5}	8.39	318.9	1.18×10^{-12}	0.996
PHB_{39}-*b*-PCL_{61} (PCL crystallization from amorphous PHB)	1.25×10^{6}	9.57×10^{4}	7.70	112.9	4.17×10^{-13}	0.994
PHB_{39}-*b*-PCL_{61} (PCL crystallization from crystalline PHB)	1.67×10^{7}	1.18×10^{5}	5.80	137.0	5.10×10^{-13}	0.996
P(HB_{72}-*ran*-CL_{28})	1.83×10^{5}	2.77×10^{5}	8.39	253.4	9.35×10^{-13}	0.997
R/S PHB-38K[2]	7.22×10^{12}	6.20×10^{5}	8.39	699.9	2.58×10^{-12}	0.990
PCL-22K[6]	3.14×10^{8}	2.10×10^{5}	7.07	230.9	8.60×10^{-13}	0.996

The Lauritzen and Hoffman theory[3] described previously can be used to fit the overall isothermal crystallization data obtained from the DSC. In this case, the equation to be applied changes with the introduction of a new parameter, i.e., the inverse of the half-crystallization time, $\tau_{50\%}$, and becomes as follows:

$$\frac{1}{\tau_{50\%}} = \frac{1}{\tau_{50\%}} exp\left[\frac{U}{R(T_c - T_0)}\right]\left[\frac{-K_g^{\tau}}{fT(T_m^0 - T_c)}\right]$$

All terms have already been described in the main text (Paragraph 3.4), except for K_g^τ which now becomes a constant that is proportional to the energy barrier for both primary nucleation and spherulitic growth. Thus, it encompasses more information than K_g^G which only refers to secondary nucleation or growth.

The experimental data of isothermal crystallization have been fitted, as described in the main text. Figures S4 through S7present examples of comparisons between the DSC experimental isotherms and the prediction of the Avrami fit (a) and the typical Avrami plot in the conversion range used to make the fit (b), obtained using the free App developed by Pérez-Camargo *et al.*[7]

In the case of PHB block crystallization in both samples the detailed results are shown in Tables S3 and S4. The fitting obtained is very for the whole range of T_c in the primary crystallization range (employing a conversion range between 3 and 20% where the free growth approximation is usually valid), and the correlation coefficient was always greater than 0.999. Also, for the value of $\tau_{50\%,}$ the values predicted by Avrami's theory are in good agreement with the experimental ones, indicating that the fits are a good representation of the experimental data until 50% conversion. Tables S3 and S4 also show the Avrami index, which assumes, on average, the value of 3, and this indicates the presence of instantaneously nucleated spherulites on average for the entire crystallization temperature range, as demonstrated by PLOM analysis.

The overall isothermal crystallization experimental data for the crystallization of the PCL block was also fitted with Avrami's theory using the free app developed by Pérez-Camargo *et al.*[7]. The detailed results are shown in Table S5 (crystalline PHB) and S6 (amorphous PHB). The fit was performed in the primary crystallization regime, and the correlation factor was always high (>0.999). The agreement is also high between the experimental and theoretical data of $\tau_{50\%}$. Tables S5 and S6 also report the Avrami

indices: it can be observed that, in the case of crystallization of PCL from amorphous and crystalline PHB, the value of n assumes, on average, the value of 3, indicative of the presence of instantaneous spherulites for all T_c.

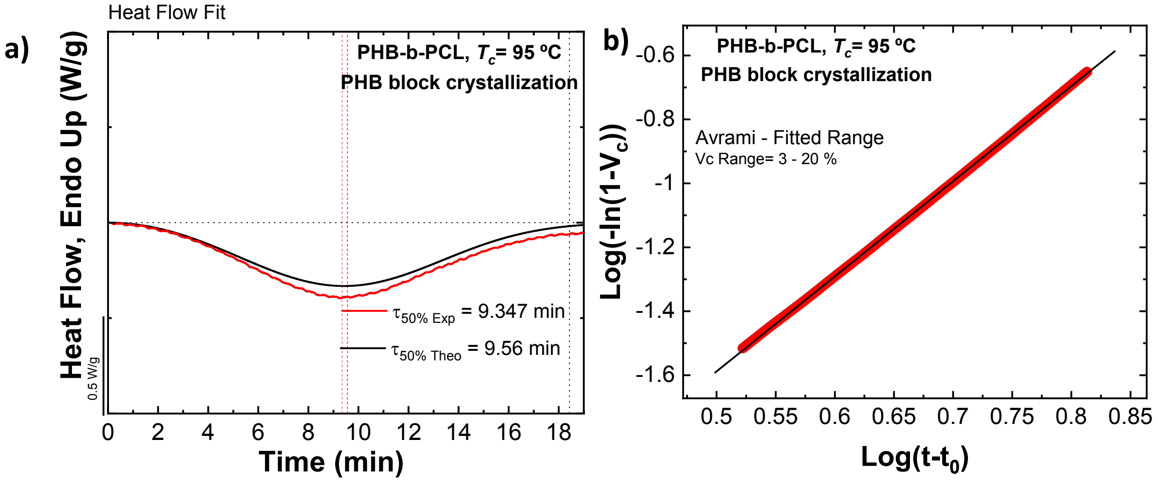

Figure S4. The Avrami fit equation using the free Origin plug-in developed by Pérez-Camargo *et al.* [7] for PHB block crystallization in PHB_{39}-*b*-PCL_{61} sample at T_c= 95 ºC.

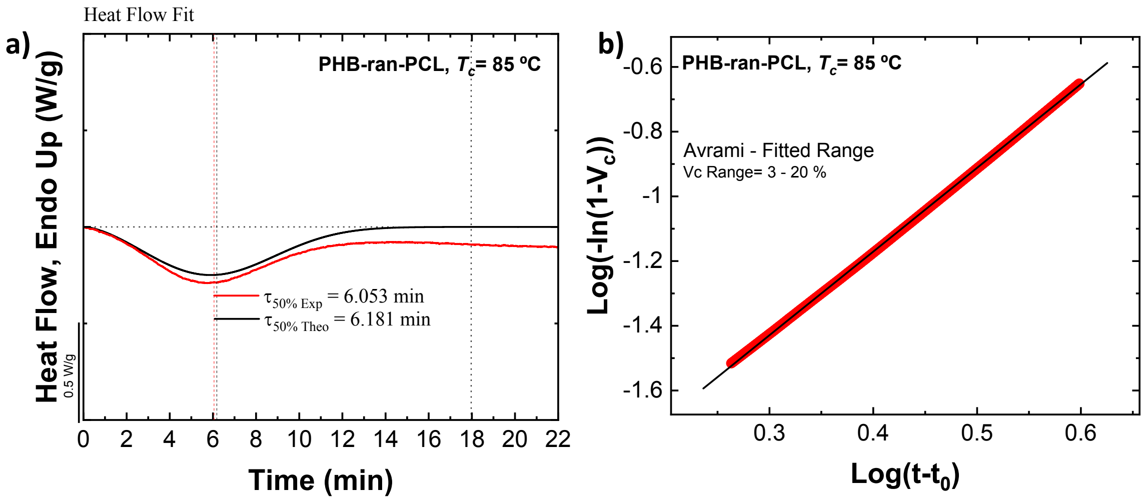

Figure S5. The Avrami fit equation using the free Origin plug-in developed by Pérez-Camargo *et al.* [7] for P(HB_{72}-*ran*-CL_{28}) at T_c= 85 ºC.

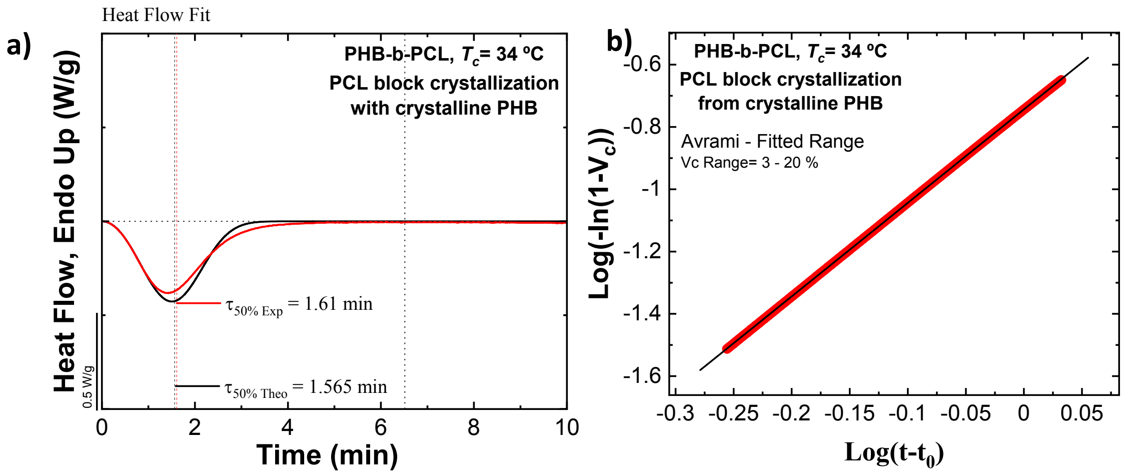

Figure S6. The Avrami fit equation using the free Origin plug-in developed by Pérez-Camargo *et al.* [7] for PCL block crystallization in PHB_{39}-*b*-PCL_{61} sample with crystalline PHB at T_c= 34 ºC.

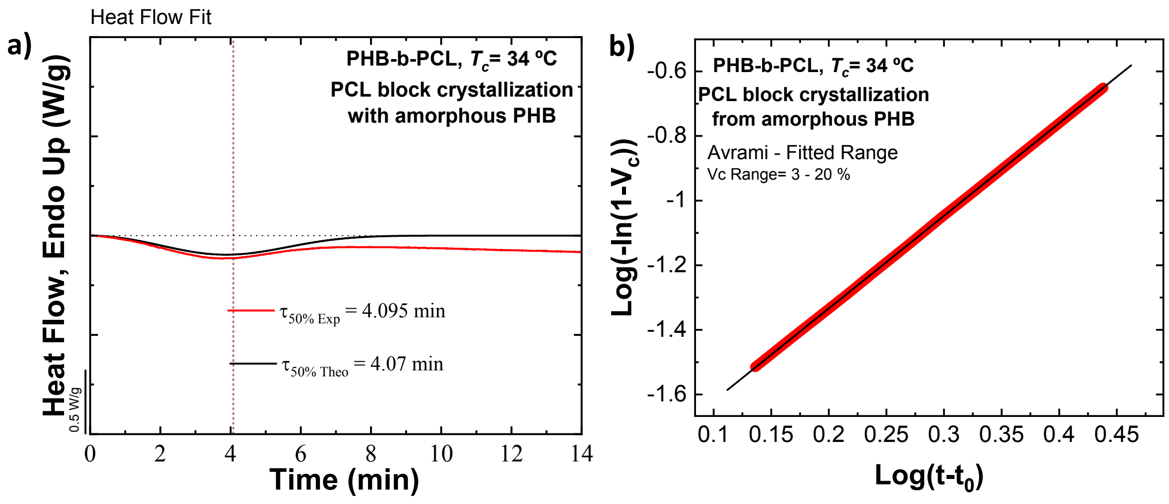

Figure S7. The Avrami fit equation using the free Origin plug-in developed by Pérez-Camargo *et al.* [7] for PCL block crystallization in PHB_{39}-*b*-PCL_{61} sample with amorphous PHB at T_c= 34 ºC.

Table S3. Avrami fitting parameters obtained from by the free App [7] for PHB crystallization in PHB_{39}-*b*-PCL_{61} sample.

T_c(ºC)	t_0(min)	$\tau_{50\%}$ Exp (min)	$\tau_{50\%\ Theo}$ (min)	n	$K^{\frac{1}{n}}$(min^{-1})	K (min^{-n})	R^2
65	0.56	2.674	2.772	2.84	0.316	3.82x10^{-2}	0.999
68	0.581	2.767	2.892	2.71	0.301	3.88x10^{-2}	0.999
70	0.653	3.172	3.214	2.96	0.274	2.18x10^{-2}	0.999
75	0.653	3.84	3.74	2.75	0.233	1.84x10^{-2}	0.999
80	0.66	4.753	4.703	2.97	0.408	7.00x10^{-2}	0.999
85	0.68	6.502	6.664	2.894	0.137	3.20x10^{-3}	0.999
90	0.685	7.47	7.884	2.62	0.266	3.13x10^{-2}	0.999
93	0.735	8.117	8.127	2.76	0.107	2.14x10^{-3}	0.999
95	0.755	9.347	9.56	2.97	0.092	8.41x10^{-4}	0.999
97	0.772	8.882	8.837	2.68	0.098	2.01x10^{-3}	0.999
100	0.78	9.097	9.143	2.58	0.094	2.30x10^{-3}	0.999
102	0.803	12.678	12.543	2.94	0.070	4.10x10^{-4}	0.999
103	0.872	13.3	13.478	3.07	0.065	2.33x10^{-4}	0.999

Table S4. Avrami fitting parameters obtained from by the free App [7] for P(HB_{72}-*ran*-CL_{28}) sample.

T_c(ºC)	t_0(min)	$\tau_{50\%}$ *Exp* (min)	$\tau_{50\%\ Theo}$ (min)	*n*	$K^{\frac{1}{n}}$(min^{-1})	K (min^{-n})	R^2
70	0.428	3.502	3.513	2.86	0.251	1.92x10^{-2}	0.999

75	0.448	3.74	3.793	2.63	0.229	$2.09x10^{-2}$	0.999
77	0.507	3.918	3.843	2.86	0.213	$5.65x10^{-2}$	0.999
80	0.613	4.712	4.787	2.62	0.181	$1.14x10^{-2}$	0.999
83	0.613	5.06	5.164	2.69	0.168	$8.37x10^{-3}$	0.999
85	0.738	6.053	6.181	2.59	0.140	$6.24x10^{-3}$	0.999
87	0.785	7.507	7.446	2.98	0.118	$1.74x10^{-}3$	0.999
90	0.95	9.749	9.708	2.71	0.088	$1.42x10^{-3}$	0.999

Table S5. Avrami fitting parameters obtained from by the free App [7] for PCL crystallization from crystalline PHB in PHB_{39}-*b*-PCL_{61} sample.

T_c(ºC)	t_0(min)	$\tau_{50\%\ Exp}$ (min)	$\tau_{50\%\ Theo}$ (min)	*n*	$K^{\frac{1}{n}}(min^{-1})$	*K* (min^{-n})	R^2
31	0.387	0.49	0.489	2.44	1.757	3.96	0.999
32	0.448	0.667	0.671	2.49	1.285	1.87	0.999
33	0.482	1.06	1.046	2.67	0.833	$6.15x10^{-1}$	0.999
34	0.495	1.61	1.565	3	0.565	$1.81x10^{-1}$	0.999
35	0.628	2.324	2.251	3.06	0.394	$5.79x10^{-2}$	0.999
36	0.872	3.365	3.309	2.9	0.266	$2.16x10^{-2}$	0.999

Table S6. Avrami fitting parameters obtained from by the free App [7] for PCL crystallization from amorphous PHB in PHB_{39}-*b*-PCL_{61} sample.

T_c(ºC)	t_0(min)	$\tau_{50\%\,Exp}$ (min)	$\tau_{50\%\,Theo}$ (min)	n	$K^{\frac{1}{n}}$(min^{-1})	K (min^{-n})	R^2
29	0.355	0.753	0.779	2.71	1.483	1.36	0.999
30	0.51	1.103	1.112	2.86	0.547	$5.12x10^{-1}$	0.999
31	0.525	1.675	1.651	3.20	0.302	$1.39x10^{-1}$	0.999
32	0.542	1.875	1.969	3.0	0.358	$1.33x10^{-1}$	0.999
33	0.577	2.495	2.277	3.1	0.283	$5.69x10^{-2}$	0.999
34	0.722	4.095	4.07	2.87	0.340	$1.24x10^{-2}$	0.999

(1) Tang, X.; Shi, C.; Zhang, Z.; Chen, E. Y. X. Toughening Biodegradable Isotactic Poly(3-Hydroxybutyrate) via Stereoselective Copolymerization of a Diolide and Lactones. *Macromolecules* **2021**, *54* (20), 9401–9409. https://doi.org/10.1021/acs.macromol.1c01199.

(2) Caputo, M. R.; Tang, X.; Westlie, A. H.; Sardon, H.; Chen, E. Y.-X.; Müller, A. J. Effect of Chain Stereoconfiguration on Poly (3-Hydroxybutyrate) Crystallization Kinetics. *Biomacromolecules* **2022**, *23* (9), 3847–3859. https://doi.org/https://doi.org/10.1021/acs.biomac.2c00682.

(3) Hoffman, J. D.; Lauritzen, J. I.; Jr. Crystallization of Bulk Polymers With Chain Folding: Theory of Growth of Lamellar Spherulites. *J. Res. Natl. Bur. Stand. Sect. A, Phys. Chem.* **1961**, *65A* (4), 297. https://doi.org/10.6028/JRES.065A.035.

(4) Pitt, C. G.; Chasalow, F. I.; Hibionada, Y. M.; Klimas, D. M.; Schindler, A. Aliphatic Polyesters. I. The Degradation of Poly (E-caprolactone) in Vivo. *J. Appl. Polym. Sci.* **1981**, *26* (11), 3779–3787. https://doi.org/https://doi.org/10.1002/app.1981.070261124.

(5) Mark, J. E. *Physical Properties of Polymers Handbook*; Springer, 2007; Vol. 1076.

(6) Fernández-Tena, A.; Pérez-Camargo, R. A.; Coulembier, O.; Sangroniz, L.; Aranburu, N.; Guerrica-Echevarria, G.; Liu, G.; Wang, D.; Cavallo, D.; Müller, A. J. Effect of Molecular Weight on the Crystallization and Melt Memory of Poly (ε-Caprolactone)(PCL). *Macromolecules* **2023**.

(7) Pérez-Camargo, R. A.; Liu, G.; Wang, D.; Müller, A. J. Experimental and Data Fitting Guidelines for the Determination of Polymer Crystallization Kinetics. *Chinese J. Polym. Sci* **2022**, *40*, 1–34.

https://doi.org/https://doi.org/10.1007/s10118-022-2724-2.
